# Emissions reduction strategy in a three-stage agrifood value chain: A dynamic differential game approach

**DOI:** 10.1371/journal.pone.0294472

**Published:** 2023-11-17

**Authors:** Huanhuan Wang, Xiaoli Fan, Qilan Zhao, Pengfei Cui

**Affiliations:** 1 School of Economics and Management, Beijing Jiaotong University, Beijing, China; 2 Department of Resource Economics and Environmental Sociology, University of Alberta, Edmonton, Alberta, Canada; 3 School of System Science, Beijing Jiaotong University, Beijing, China; UCL: University College London, UNITED KINGDOM

## Abstract

Agrifood systems account for 31% of global greenhouse gas emissions. Substantial emissions reduction in agrifood systems is critical to achieving the temperature goal set by the Paris Agreement. A key challenge in reducing GHG emissions in the agrifood value chain is the imbalanced allocation of benefits and costs associated with emissions reduction among agrifood value chain participants. However, only a few studies have examined agrifood emissions reduction from a value chain perspective, especially using dynamic methods to investigate participants’ long-term emissions reduction strategies. This paper helps fill this gap in the existing literature by examining the impact of collaborations among agrifood value chain participants on correcting those misallocations and reducing emissions in agrifood systems. We develop a dynamic differential game model to examine participants’ long-term emissions reduction strategies in a three-stage agrifood value chain. We use the Hamilton-Jacobi-Bellman equation to derive the Nash equilibrium emissions reduction strategies under non-cooperative, cost-sharing, and cooperative mechanisms. We then conduct numerical analysis and sensitivity analysis to validate our model. Our results show that collaboration among value chain participants leads to higher emissions reduction efforts and profits for the entire value chain. Specifically, based on our numerical results, the cooperative mechanism results in the greatest emissions reduction effort by the three participants, which leads to a total that is nearly three times higher than that of the non-cooperative mechanism and close to two times higher than the cost-sharing mechanism. The cooperative mechanism also recorded the highest profits for the entire value chain, surpassing the non-cooperative and cost-sharing mechanisms by around 37% and 16%, respectively. Our results provide valuable insights for policymakers and agrifood industry stakeholders to develop strategies and policies encouraging emissions reduction collaborations in the agrifood value chain and reduce emissions in the agrifood systems.

## 1. Introduction

Agrifood systems account for 31% of total greenhouse gas (GHG) emissions [1]. Substantial emissions reduction in agrifood systems is critical to achieving the temperature goal set by the Paris Agreement. A key challenge in reducing GHG emissions in the agrifood value chain is the imbalanced allocation of benefits and costs associated with emissions reduction among agrifood value chain participants. Along the agrifood value chain, emissions mainly occur at the upstream farm-gate production stage. According to FAO [2], farm gate emissions and land use change represent about 65% of the total emissions of the agrifood system. Nonetheless, most value added happens at the processing and packing stage, where margins are the highest. In response to increasing consumer demand for low-emission agrifood products, many international agrifood companies–primarily processors and packers (e.g., Danone, Nestle, General Mills Inc.)–have set company-specific emissions reduction targets [3]. To achieve emissions reduction targets, agrifood processors and packers likely have to rely on upstream farmers to adopt lower-emission farming practices. However, there is a lack of incentive for farmers to change their farming practices. For each dollar consumers spend on food, farmers only receive a small share (14.5%) [4]. Additionally, changing practices to reduce emissions is associated with high costs and risks that cause significant burdens to farmers. The misalignment of objectives among agrifood value chain participants poses a severe challenge for reducing emissions in the agrifood systems. Effective collaboration among value chain participants is necessary for the agrifood industry to meet consumer demand for low-emission products while ensuring associated benefits and costs to produce such products are equitably born by value chain participants.

The objective of our paper is to study the impact of collaboration on agrifood value chain participants’ long-term strategic behavior in reducing emissions. Previous studies [5–12] have identified several common collaborative mechanisms in the agricultural sector: non-cooperation [6,13–15], cost-sharing [14,16–18], and cooperation [13,19–21]. These collaborative mechanisms represent various degrees of collaboration among value chain participants, which might lead to value chain participants exhibiting different strategic behaviors. Therefore, this paper also aims to evaluate the effectiveness of these collaborative mechanisms on value chain participants’ emissions reduction efforts and the resulting profit of the overall value chain, which will be valuable to policymakers and stakeholders in the agrifood industry.

There has been a growing body of literature on agrifood value chain to study how coordination and collaboration can help achieve goals of agrifood value chain. Reviews of this literature can be found in several studies [22–24]. Our research contributes to this literature in two ways. First, studies in this literature examined agrifood value chain collaboration on a diverse range of topics including maintaining product freshness, contract practices, the role of trust, achieving United Nations Sustainable Development Goals, among others. However, only a few studies have directly examined emissions reduction collaboration. Our research therefore adds to the existing literature by studying collaboration among agrifood value chain participants on emissions reduction.

Second, our research also contributes to the agrifood value chain collaboration literature by applying a new research method–dynamic differential games–to study collaboration among agrifood value chain participants. This approach has been widely used by researchers in the fields of engineering, political science, and marketing to analyze decision-making processes involving multiple players and unfold over time [25–33]. But we are not aware of any previous studies in the agrifood value chain collaboration literature that have applied the dynamic differential game approach. The most popular method used in the agrifood value chain collaboration literature is case studies [34–37]. Other methods, such as statistical modeling [38,39], mathematical programming [40,41], analytical model [42–44], and qualitative analysis [45,46] have also been used. Although these studies provided valuable insights into different aspects of agrifood value chain collaboration, they cannot quantitatively capture the complex dynamics of strategic interactions among value chain participants. Simulation methods [47,48] are capable of tackling the dynamic aspect and strategic interaction aspect simultaneously and have been employed by researchers in many fields to model the dynamic aspects of system operations [49–52]. While simulation methods can capture intricate system dynamics, they may not explicitly capture the depth of strategic decision-making like dynamic differential games. The dynamic differential game model we utilized effectively captures the dynamic nature of emissions reduction processes under different collaborative mechanisms, allowing for the examination of continuous-time control variables. The ability of our model to study the long-term strategic behaviors of the value chain participants is of utmost importance, as reducing emissions in agrifood systems often entails adopting new farm management practices that may take years to fully implement [53]. Our research also complements previous studies that used simulation methods to model dynamic strategic behaviors in the agrifood value chain.

Our research also contributes to the general supply management literature by extending the application of the dynamic differential game model from two-level supply chains to three-stage agricultural value chains. A large body of research, exemplified by the work of Ma et al. [54] and Jian et al. [55], has explored emissions reduction in the context of two-level supply chains by employing game theory models, such as Stackelberg and differential games, to analyze participant behaviors [25,56]. Nonetheless, this body of literature largely neglects the dynamic analysis of emissions reduction in three-stage value chains, with a notable gap in the context of the agrifood sector. Even within the existing research on emissions reduction in agrifood systems, only a few studies tackle this issue from a three-stage value chain perspective [57–59]. Moreover, there is even less research on the long-term collaboration of the three participants and its impact on emissions reduction in agrifood systems [12]. It is unclear whether value chain collaboration can effectively reduce GHG emissions and how the potential gains from emissions reduction will be distributed among agrifood value chain participants. In this context, our research employs dynamic differential game approach to fill this gap, examining the impact of emissions reduction collaboration within a three-stage agrifood value chain that consists of a producer, packer, and retailer.

Finally, although our model is developed to study the problem of emissions reduction collaboration in the agrifood value chain, it can be easily adapted to explore other issues requiring collaboration among value chain participants within this industry. Many issues in agriculture involve multiple players in a long-term game, making the research problem dynamic. Therefore, our dynamic model is more appropriate for such research than a limited static approach. For instance, our model can investigate how different stakeholders collaborate to enhance transparency and traceability for food safety over time. Similarly, it can also examine how collaboration at various stages minimizes waste during production, processing, or retail in response to changing scenarios. Additionally, our model can be extended to analyze emissions reduction collaborations in other industries that exhibit a similar three-stage value chain, especially in cases where dynamic collaboration among value chain participants is essential for achieving emissions reduction goals. For example, in the logistics industry, dynamic collaboration among stakeholders such as manufacturers, transporters, and retailers is essential to develop more sustainable logistics solutions. Likewise, within the beef sector, partnerships among ranchers, packers, and retailers can be analyzed to formulate profitable emissions reduction strategies over time. In the food and beverage industry, the three-tier value chain consists of raw material supplier, food processing company, and retailer, typically collaborating to meet shared low-carbon objectives.

The remainder of this study is organized as follows: Section 2 presents the dynamic differential game model of emissions reduction in the agrifood value chain under three collaborative mechanisms. In Section 3, we derive our theoretical results, including each agrifood value chain participant’s optimal emissions reduction strategy and the resulting maximized profit. We further compare the results arising from the three different collaborative mechanisms. In Section 4, we conduct numerical analysis and sensitivity analysis to validate the models. Finally, Section 5 concludes the study and discusses the policy implications of our research, potential limitations, and suggestions for future research on GHG emissions reduction in agrifood systems from the perspective of value chain participants’ collaboration.

## 2. Dynamic differential game model of emissions reduction collaboration

**Fig 1:**
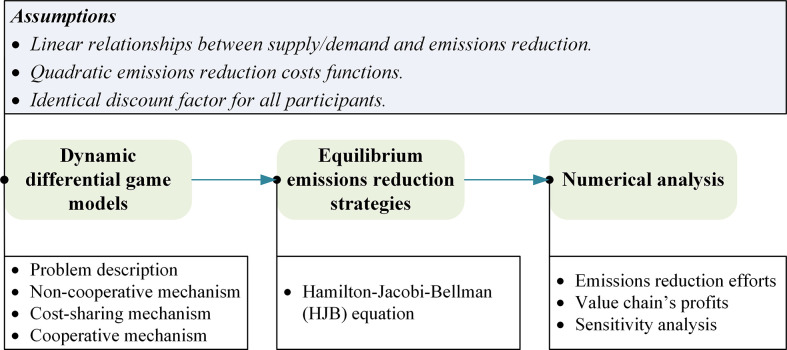
The methodology of this study.

In this section, we start with a comprehensive description of the research problem and then address the assumptions. Based on this foundation, we construct dynamic differential game models under three collaborative mechanisms mentioned above. Fig 1 illustrates the methodology of this study.

### 2.1 Problem description

**Fig 2:**
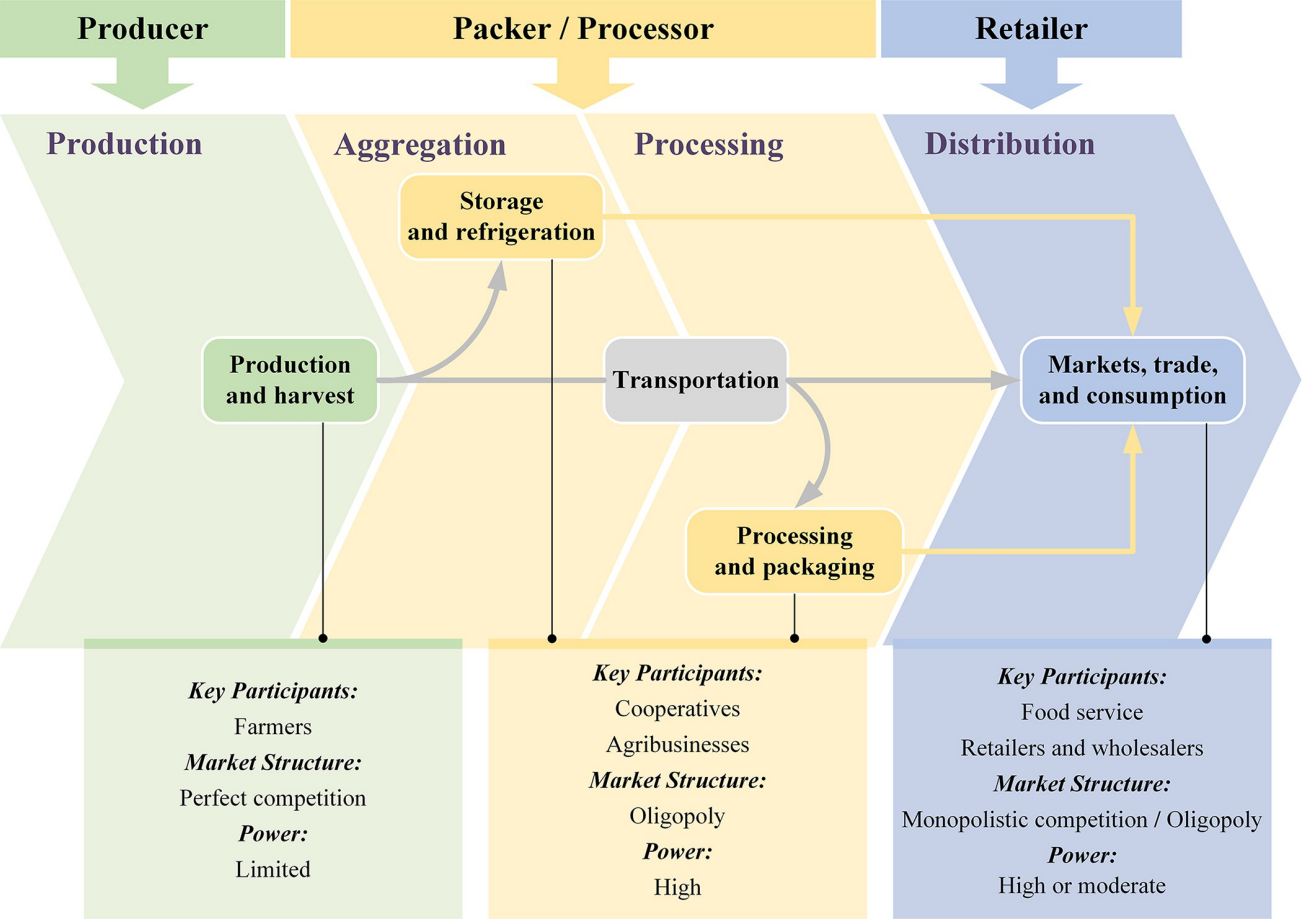
Key steps and power dynamics along the agrifood value chain. Source: Adapted from Puri (2014) [61]; FAO (2014) [62]; FAO and UNDP (2020) [63]; FAO (2022) [58].

In an agricultural context, an agrifood value chain can be defined as a set of participants and activities involved in taking an agricultural product from production to its final consumption, with value being added at each stage [60]. Agrifood value chains encompass four main functions–production (cultivation, raising, and harvesting), aggregation (storage and refrigeration), processing (processing and packaging), and distribution (markets, trade, and consumption)–transportation is integral throughout the entire chain, and various interconnected participants are engaged at each step [58]. Fig 2 illustrates the essential steps and power dynamics in an agrifood value chain from harvest to market.

As depicted in Fig 2, in the agrifood value chain, the producer takes charge of the production stages, and the packer predominantly focuses on the aggregation and processing phases. The retailer then assumes a central role towards the end of the value chain. Additionally, by comparing the market structure of the different segments and the power of the participants involved, we observe that the packer occupies a dominant position in the agrifood value chain and has a significant impact on its operations. The packer sets standards and quality requirements, controls value addition and branding, and can shift market dynamics by prioritizing certain crops. Moreover, in packer-dominated value chains, retailers have a certain degree of negotiation power due to the monopolistic competition, enabling them to set terms and influence prices. However, owing to fragmentation and the absence of collective negotiation mechanisms, many producers often find themselves in a passive position, typically accepting requirements from the packers. Despite the agrifood value chain having various stages and distinct power dynamics, the producer, packer, and retailer all play crucial roles in its sustainable functioning.

To investigate the emissions reduction strategies of the participants under different collaborative mechanisms, we consider a three-stage agrifood value chain consisting of a producer (*s*), a processor or packer (*m*), and a retailer (*r*). We focus on the most common structure of the agrifood vertical chain where the packer dominates the value chain, i.e., the packer has the highest visibility in the value chain and benefits the most from the overall product emissions reduction.

All three participants can invest in emissions reduction efforts to lower the GHG emissions of the agrifood value chain and the emissions associated with the final agrifood product. The producer (*s*) is responsible for reducing the emissions on agricultural land (e.g., growing crops, raising livestock), which is the largest source of GHG emission along the agrifood value chain. The retailer can help reduce emissions by promoting low-emission products to increase consumer demand for these products. The packer can directly reduce emissions that occur during the packing stage. The packer captures the largest benefits of emissions reduction associated with the final product, and therefore has the incentive to encourage emissions reduction collaborations among value chain participants.

**Fig 3:**
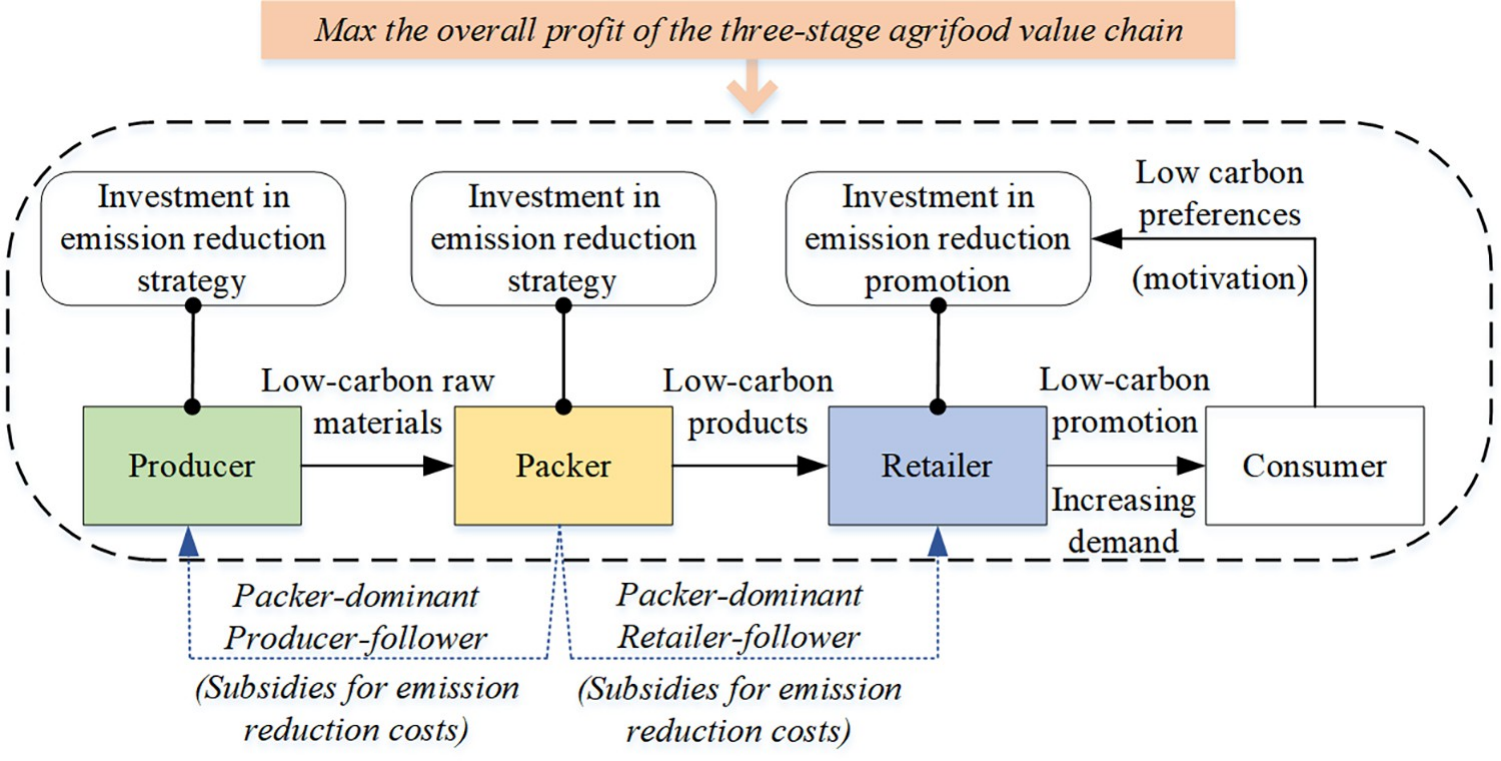
Collaborative mechanisms of GHG emissions reduction in the agrifood value chain.

We consider three collaborative mechanisms of GHG emissions reduction of the three participants in the agrifood value chain. In Fig 3, the flow chart in the dashed box indicates the operation of the agrifood value chain under the non-cooperative mechanism. The producer, packer, and retailer make independent decisions to choose the optimal emissions reduction strategy that maximizes their respective profit. Under the cost-sharing mechanism, the packer provides subsidies (blue dotted arrows) to the producer and the retailer to offset their emissions reduction costs. Under the cooperative mechanism (orange bar on top), the three participants jointly determine the emissions reduction strategies to maximize the aggregated profit of all participants involved in the agrifood value chain.

Given that emissions reduction collaboration is a dynamic game process of multiple participants over a long period, this paper adopts a dynamic differential game approach [64]. Differential game provides an effective tool to analyze the emissions reduction strategies employed by the participants and the overall profit of the agrifood value chain. These games are typically characterized by continuous-time control variables, with the dynamics of the system described by a set of differential equations.

The emissions reduction in the agrifood value chain is a dynamic process [65] that is influenced by the emissions reduction efforts of the producer (*a*_*s*_(*t*)), packer (*a*_*m*_(*t*)), and retailer (*a*_*r*_(*t*)), as well as the stock of emissions reduction achieved in the final product in the current period. This dynamic process can be represented by a differential equation:

$$\dot{q}(t)=\varepsilon a_{s}(t)+\delta a_{m}(t)+\gamma a_{r}(t)-\sigma q(t)$$ (1)

where *q*(*t*) represents the product emissions reduction at time *t*. The parameters *ε* and *δ* (both>0) represent the coefficients of producer and packer emissions reduction efforts on product emissions reduction, respectively. We use *γ* (>0) to denote the coefficient of the impact of the retailer’s low-emission promotion efforts on *q*(*t*). Additionally, considering that the emissions reduction equipment will deteriorate over time, we introduce a natural decay rate *σ* (>0) of emissions reduction associated with the final product.

We assume that the emissions reduction costs for the producer, packer and retailer are a quadratic function of their respective emissions reduction efforts [66]. Additionally, the costs of low-emission promotion for retailers are a quadratic function of the low-emission promotion efforts. Specifically, the emissions reduction costs of producer *s*, packer *m*, and the low-emission promotion costs of retailer *r* at time *t* can be expressed as:

$$C(a_{s}(t))=\frac{\mu_{s}}{2}{\left[a_{s}(t)\right]}^{2}$$ (2)

$$C(a_{m}(t))=\frac{\mu_{m}}{2}{\left[a_{m}(t)\right]}^{2}$$ (3)

$$C(a_{r}(t))=\frac{\mu_{r}}{2}{\left[a_{r}(t)\right]}^{2}$$ (4)

where *μ*_*s*_, *μ*_*m*_ and *μ*_*r*_ denote the coefficients of emissions reduction costs for producer *s*, packer *m*, and low-emission promotion costs for retailer *r*, respectively. *C*(*a*_*s*_(*t*)), *C*(*a*_*m*_(*t*)) and *C*(*a*_*r*_(*t*)) represent the emissions reduction costs for producer *s*, packer *m*, and low-emission promotion costs for retailer *r* at time *t*, respectively.

We assume a linear relationship between product supply/demand and product emissions reduction at time *t* [67,68], as shown in the following equation:

$$Q(q(t),t)=Q_{0}+\alpha (q_{0}+q(t))$$ (5)

$$D(q(t),t)=D_{0}+\varphi (q_{0}+q(t))$$ (6)

where *Q*_0_≥0 is a constant indicating the initial supply of the agrifood product. *α*>0 is the coefficient of the effect of product emissions reduction on product supply. *D*_0_≥0 is a constant indicating the initial demand for the agricultural product. *φ*>0 is the coefficient of the effect of product emissions reduction on product demand.

We assume that the discount factor *ρ* (>0) is identical for the producer, packer, and retailer. The producer, packer, and retailer are considered to be rational decision-makers who seek the optimal emissions reduction strategies that maximize their respective profits.

This paper mainly focuses on the emissions reduction strategies of the three participants and the aggregated profit of the agrifood value chain under the abovementioned collaborative mechanisms (non-cooperative, cost-sharing, and cooperative mechanisms). Hence, we do not consider factors such as inventory costs and out-of-stock costs across multiple levels of the supply chain, as well as the impact of other factors on demand, e.g., price. S1 Table describes the parameters used in our model.

### 2.2 Non-cooperative mechanism

Under the non-cooperative mechanism, the producer, packer, and retailer engage in an infinite-horizon simultaneous game, making decisions independently without explicit coordination or contracts. At each period *t*, the three participants simultaneously make decisions on their emissions reduction efforts based on their expectations of the other two participants’ emissions reduction efforts. Each of the three participants independently makes its emissions reduction effort decision to maximize its own profit. Denote the emissions reduction strategies of the producer, packer and retailer as *a*_*s*1_(*t*), *a*_*m*1_(*t*), and *a*_*r*1_(*t*), respectively.

The profit function of the producer is correlated with the supply of the product.

$$H_{s1}(a_{s1}(t))=\int_{0}^{\infty }e^{-\rho t}\{p_{s}Q(q(t),t)-\frac{\mu_{s}}{2}{\left[a_{s1}(t)\right]}^{2}\}dt$$ (7)

The profit function of the packer is associated with the demand for the product.

$$H_{m1}(a_{m1}(t))=\int_{0}^{\infty }e^{-\rho t}\{p_{m}D(q(t),t)-\frac{\mu_{m}}{2}{\left[a_{m1}(t)\right]}^{2}\}dt$$ (8)

The profit function of the retailer is also related to the demand for the product.


$$H_{r1}(a_{r1}(t))=\int_{0}^{\infty }e^{-\rho t}\{p_{r}D(q(t),t)-\frac{\mu_{r}}{2}{\left[a_{r1}(t)\right]}^{2}\}dt$$ (9)

### 2.3 Cost-sharing mechanism

In the agricultural sector, packers and producers or retailers typically enter into contracts to delineate the specific terms of their business relationship. These contracts are taken to coordinate supply chain members and foster better cooperative relationships [22]. One primary focus of these contracts, especially concerning cost-sharing, pertains to the allocation of expenses such as production, transportation, storage, marketing and promotion, and any particular agricultural practices that need to be adhered to. Since consumers are willing to pay a high premium for low-emission agrifood products, the packer is incentivized to sign contracts with the producer and retailer to collaborate on emissions reduction. In a cost-sharing contract, producers agree to grow crops using specified techniques as guided by the packer, and retailers promise robust marketing efforts for the product, both on the basis that the packer shares the associated costs. This collaboration relies on the packer bearing a portion of the costs, making it the leader in the partnership.

We consider this scenario as the cost-sharing mechanism, which involves the value chain leader sharing the costs incurred in implementing emissions reduction measures for followers. To encourage the producer to actively provide low-emission raw materials, the packer can offer to share a certain percentage of the emissions reduction costs incurred by the producer, denoted as *β*(*t*). Similarly, to encourage the retailer to actively promote low-emission products, the packer also offers to share a certain percentage of the retailer’s emissions reduction cost, denoted as *θ*(*t*).

Under this cost-sharing mechanism, the packer (dominant) engages in a two-stage Stackelberg game with the producer (follower) and retailer (follower). In the first stage, the packer decides its own emissions reduction effort and the optimal cost-sharing ratios to offer to the producer and the retailer to incentivize them to actively collaborate in emissions reduction. When making those decisions, the packer takes into account how its decisions will affect the producer’s and retailer’s emissions reduction efforts in the second stage. In the second stage, the producer and the retailer decide their optimal emissions reduction efforts, treating the packer’s emissions reduction effort and cost-sharing ratios as given. We assume that the game is played in an infinite time horizon. Denote the emissions reduction strategies of the packer, producer, and retailer as *a*_*m*2_(*t*), *a*_*s*2_(*t*), and *a*_*r*2_(*t*).

The profit function of the packer is

$$H_{m2}(a_{m2}(t),{a_{s2}}^{*}(t),{a_{r2}}^{*}(t),\beta (t),\theta (t))=\int_{0}^{\infty }e^{-\rho t}\{p_{m}D(q(t),t)-\frac{\mu_{m}}{2}{\left[a_{m2}(t)\right]}^{2}-\beta (t)\frac{\mu_{s}}{2}{\left[{a_{s2}}^{*}(t)\right]}^{2}-\theta (t)\frac{\mu_{r}}{2}{\left[{a_{r2}}^{*}(t)\right]}^{2}\}dt$$ (10)

The profit function of the producer is

$$H_{s2}(a_{s2}(t),\beta (t))=\int_{0}^{\infty }e^{-\rho t}\{p_{s}Q(q(t),t)-(1-\beta (t))\frac{\mu_{s}}{2}{\left[a_{s2}(t)\right]}^{2}\}dt$$ (11)

The profit function of the retailer is

$$H_{r2}(a_{r2}(t),\theta (t))=\int_{0}^{\infty }e^{-\rho t}\{p_{r}D(q(t),t)-(1-\theta (t))\frac{\mu_{r}}{2}{\left[a_{r2}(t)\right]}^{2}\}dt$$ (12)

### 2.4 Cooperative mechanism

Multi-stakeholder collaboration is crucial to achieving efficient supply chain operations and sustainable industry development. For example, the global beef industry is experiencing a transformative shift towards sustainability, driven by both environmental imperatives and stakeholder demands. Non-profit collaborative organizations like the Global, U.S., and Canadian Roundtables for Sustainable Beef, are pioneering this movement, leveraging expertise from various nations to promote responsible practices across the beef value chain. Recognizing the significance of climate change and the urgent need for sustainable industry practices, various members of the roundtable collaboratively set shared goals and standards for emissions reduction, ensuring alignment with all stakeholders. Similarly, major corporations like McDonald’s and Cargill are spearheading emissions reduction within their supply chains. Recognizing the intertwined nature of the industry, these entities provide essential technical support and resources to their suppliers. Such collaborative efforts not only underscore the importance of sustainability but also illustrate how fostering environmentally conscious practices can yield long-term profitability and foster stronger business relationships.

The incentives driving the cooperative framework mainly include: non-profit organizations that foster collaborative efforts among stakeholders; funding from governments or businesses that support collaborative initiatives for ecological objectives; leading participants committed to cultivating an environmentally responsible reputation; followers eager to maintain a robust partnership with the key player; and an increasing consumer demand for eco-friendly agricultural products. In the case of a cooperative game, for long-term and sustainable operation of the value chain, the packer, the producer and the retailer jointly determine the emissions reduction efforts $a_{m}^{c},a_{s}^{c}$ and $a_{r}^{c}$ to maximize the aggregated profit of all participants involved in the agrifood value chain. The profit function of the agrifood value chain, denoted by $H_{w}^{c}$, can be expressed as follows:

$$H_{w}^{c}(a_{m}^{c}(t),a_{s}^{c}(t),a_{r}^{c}(t))=\int_{0}^{\infty }e^{-\rho t}\{(p_{m}+p_{r})D(q(t),t)+p_{s}Q(q(t),t)-\frac{\mu_{m}}{2}{\left[a_{m}^{c}(t)\right]}^{2}-\frac{\mu_{s}}{2}{\left[a_{s}^{c}(t)\right]}^{2}-\frac{\mu_{r}}{2}{\left[a_{r}^{c}(t)\right]}^{2}\}dt$$ (13)

## 3. Results and discussion

We use the Hamilton-Jacobi-Bellman (HJB) equation to solve the game of the three-stage agrifood value chain under the three collaborative mechanisms. The HJB equation is a partial differential equation (PDE) that arises when seeking a value function, which represents the optimal cost (or reward) for each participant, given the current state of the system. By using the HJB equation, we obtain the equilibrium emissions reduction strategies of the packer, producer and retailer, the trajectory of emissions reduction, and the aggregated profit of the agrifood value chain.

### 3.1. Equilibrium emissions reduction strategy under the non-cooperative mechanism

**Proposition 1.** Under the non-cooperative mechanism, the emissions reduction strategies of the packer, producer, and retailer are $\left({a_{s1}}^{*},{a_{m1}}^{*},{a_{r1}}^{*}\right)$. The optimal trajectory of product emissions reduction is $q(t)=z-(z-q_{0})e^{-\sigma t}.$ *The optimal value function of present profit for the three participants are* $H_{s1}^{*}(q)=e^{-\rho t}{Y_{s1}(q)}^{*},H_{m1}^{*}(q)=e^{-\rho t}{Y_{m1}(q)}^{*},H_{r1}^{*}(q)=e^{-\rho t}{Y_{r1}(q)}^{*}$.

**Proof.** See S1 File.


$${a_{s1}}^{*}=\frac{\varepsilon p_{s}\alpha }{\mu_{s}(\rho +\sigma )}$$

$${a_{m1}}^{*}=\frac{\delta p_{m}\varphi }{\mu_{m}(\rho +\sigma )}$$

$${a_{r1}}^{*}=\frac{\gamma p_{r}\varphi }{\mu_{r}(\rho +\sigma )}$$

$$z=\frac{\varepsilon^{2}p_{s}\alpha }{\sigma \mu_{s}(\rho +\sigma )}+\frac{\delta^{2}p_{m}\varphi }{\sigma \mu_{m}(\rho +\sigma )}+\frac{\gamma^{2}p_{r}\varphi }{\sigma \mu_{r}(\rho +\sigma )}$$

$${Y_{s1}(q)}^{*}=\frac{p_{s}\partial }{\rho +\sigma }q+\frac{p_{s}Q_{0}}{\rho }+\frac{\varepsilon^{2}{p_{s}}^{2}\alpha^{2}}{{2\rho \mu }_{s}{\left(\rho +\sigma \right)}^{2}}+\frac{\delta^{2}\alpha \varphi p_{s}p_{m}}{{\rho \mu }_{m}{\left(\rho +\sigma \right)}^{2}}+\frac{\gamma^{2}\alpha \varphi p_{s}p_{r}}{\rho \mu_{r}{\left(\rho +\sigma \right)}^{2}}$$

$${Y_{m1}(q)}^{*}=\frac{p_{m}\varphi }{\rho +\sigma }q+\frac{p_{m}D_{0}}{\rho }+\frac{\varepsilon^{2}\varphi {\alpha p}_{m}p_{s}}{\rho \mu_{s}{\left(\rho +\sigma \right)}^{2}}+\frac{\delta^{2}\varphi^{2}{p_{m}}^{2}}{{2\rho \mu }_{m}{\left(\rho +\sigma \right)}^{2}}+\frac{\gamma^{2}{\varphi^{2}p}_{m}p_{r}}{\rho \mu_{r}{\left(\rho +\sigma \right)}^{2}}$$

$${Y_{r1}(q)}^{*}=\frac{p_{r}\varphi }{\rho +\sigma }q+\frac{p_{r}D_{0}}{\rho }+\frac{\varepsilon^{2}{\alpha \varphi p}_{r}p_{s}}{\rho \mu_{s}{\left(\rho +\sigma \right)}^{2}}+\frac{\delta^{2}\varphi^{2}p_{r}p_{m}}{{\rho \mu }_{m}{\left(\rho +\sigma \right)}^{2}}+\frac{\gamma^{2}{{p_{r}}^{2}\varphi }^{2}}{2\rho \mu_{r}{\left(\rho +\sigma \right)}^{2}}$$

### 3.2. Equilibrium emissions reduction strategy under the cost-sharing mechanism

#### Proposition 2

Under the packer-led cost-sharing mechanism, the feedback Stackelberg equilibrium strategies of the packer, producer, and retailer regarding the degree of emissions reduction effort are ($\left({a_{m2}}^{*},\beta^{*},\theta^{*}),{a_{s2}}^{*},{a_{r2}}^{*}\right)$. *The optimal trajectory of product emissions reduction is* $q(t)=\omega -(\omega -q_{0})e^{-\sigma t}.$ *The optimal value function of present profit for the three participants are* $H_{s2}^{*}(q)=e^{-\rho t}{Y_{s2}(q)}^{*},H_{r2}^{*}(q)=e^{-\rho t}{Y_{r2}(q)}^{*},H_{m2}^{*}(q)=e^{-\rho t}{Y_{m2}(q)}^{*}$.

**Proof.** See S1 File.


$${a_{s2}}^{*}=\frac{\varepsilon (2\varphi p_{m}+\alpha p_{s})}{2\mu_{s}(\rho +\sigma )}$$

$${a_{r2}}^{*}=\frac{\gamma \varphi (2p_{m}+p_{r})}{2\mu_{r}(\rho +\sigma )}$$

$${a_{m2}}^{*}=\frac{\delta \varphi p_{m}}{\mu_{m}(\rho +\sigma )}$$

β*={2φpm−αps2φpm+αps0whenφpm≥αps2whenφpm<αps2

θ*={2pm−pr2pm+pr0whenpm≥pr2whenpm<pr2

$$\omega =\frac{\varepsilon^{2}(2\varphi p_{m}+\alpha p_{s})}{2{\sigma \mu }_{s}(\rho +\sigma )}+\frac{\gamma^{2}\varphi (2p_{m}+p_{r})}{2\sigma \mu_{r}(\rho +\sigma )}+\frac{\delta^{2}\varphi p_{m}}{\sigma \mu_{m}(\rho +\sigma )}$$

$${Y_{s2}(q)}^{*}=\frac{p_{s}\alpha }{\rho +\sigma }q+\frac{p_{s}Q_{0}}{\rho }+\frac{\delta^{2}\alpha \varphi p_{s}p_{m}}{\rho \mu_{m}{\left(\rho +\sigma \right)}^{2}}+\frac{\varepsilon^{2}\alpha p_{s}(2\varphi p_{m}+\alpha p_{s})}{4\rho \mu_{s}{\left(\rho +\sigma \right)}^{2}}+\frac{\gamma^{2}\alpha \varphi p_{s}(2p_{m}+p_{r})}{2\rho \mu_{r}{\left(\rho +\sigma \right)}^{2}}$$

$${Y_{r2}(q)}^{*}=\frac{p_{r}\varphi }{\rho +\sigma }q+\frac{p_{r}D_{0}}{\rho }+\frac{\delta^{2}\varphi^{2}p_{r}p_{m}}{\rho \mu_{m}{\left(\rho +\sigma \right)}^{2}}+\frac{\varepsilon^{2}\varphi p_{r}(2\varphi p_{m}+\alpha p_{s})}{2\rho \mu_{s}{\left(\rho +\sigma \right)}^{2}}+\frac{\gamma^{2}\varphi^{2}p_{r}(2p_{m}+p_{r})}{4\rho \mu_{r}{\left(\rho +\sigma \right)}^{2}}$$

$${Y_{m2}(q)}^{*}=\frac{p_{m}\varphi }{\rho +\sigma }q+\frac{p_{m}D_{0}}{\rho }+\frac{\delta^{2}\varphi^{2}{p_{m}}^{2}}{2\rho \mu_{m}{\left(\rho +\sigma \right)}^{2}}+\frac{\varepsilon^{2}{\left(2\varphi p_{m}+\alpha p_{s}\right)}^{2}}{8\rho \mu_{s}{\left(\rho +\sigma \right)}^{2}}+\frac{\gamma^{2}\varphi^{2}{\left(2p_{m}+p_{r}\right)}^{2}}{8\rho \mu_{r}{\left(\rho +\sigma \right)}^{2}}$$

### 3.3. Equilibrium emissions reduction strategy under the cooperative mechanism

#### Proposition 3

Under the cooperative mechanism, the feedback Stackelberg equilibrium strategies of the packer, producer, and retailer are $\left({a_{m}^{c}}^{*},{a_{s}^{c}}^{*},{a_{r}^{c}}^{*}\right)$. The optimal trajectory of product emissions reduction is ${{q(t)}^{c}}^{*}=v-(v-q_{0})e^{-\sigma t}.$ *The optimal value function of present profit for the agrifood value chain is* ${H_{w}^{c}}^{*}(q)=e^{-\rho t}{Y_{w}(q)}^{*}$.

**Proof.** See S1 File.


$${a_{m}^{c}}^{*}=\frac{\delta [(p_{m}+p_{r})\varphi +p_{s}\alpha ]}{\mu_{m}(\rho +\sigma )}$$

$${a_{s}^{c}}^{*}=\frac{\varepsilon [(p_{m}+p_{r})\varphi +p_{s}\alpha ]}{\mu_{s}(\rho +\sigma )}$$

$${a_{r}^{c}}^{*}=\frac{\gamma [(p_{m}+p_{r})\varphi +p_{s}\alpha ]}{\mu_{r}(\rho +\sigma )}$$

$$v=\frac{\delta^{2}[(p_{m}+p_{r})\varphi +p_{s}\alpha ]}{\sigma \mu_{m}(\rho +\sigma )}+\frac{\varepsilon^{2}[(p_{m}+p_{r})\varphi +p_{s}\alpha ]}{{\sigma \mu }_{s}(\rho +\sigma )}+\frac{\gamma^{2}[(p_{m}+p_{r})\varphi +p_{s}\alpha ]}{\sigma \mu_{r}(\rho +\sigma )}$$

$${Y_{w}(q)}^{*}=\frac{(p_{m}+p_{r})\varphi +p_{s}\alpha }{\rho +\sigma }q+\frac{(p_{m}+p_{r})D_{0}+p_{s}Q_{0}}{\rho }+\frac{{\left[(p_{m}+p_{r})\varphi +p_{s}\alpha \right]}^{2}}{2\rho {\left(\rho +\sigma \right)}^{2}}\left(\frac{\delta^{2}}{\mu_{m}}+\frac{\varepsilon^{2}}{\mu_{s}}+\frac{\gamma^{2}}{\mu_{r}}\right)$$

### 3.4. Discussion

From the feedback Nash equilibrium strategies we derived for the three collaborative mechanisms, we observe both similarities and differences in the strategic behaviors of the three value chain participants. We first summarize the similarities in Inference 1 and then discuss the differences in Proposition 4.

#### Inference 1

Under all three collaborative mechanisms, we observe that as the marginal profits of the packer, producer, and retailer (*p*_*s*_, *p*_*m*_, *p*_*r*_) increase, they are willing to put more emissions reduction effort into increasing their profits. When their emissions reduction efforts are easily translated into an increase in product emissions reduction (i.e., the coefficients of the emissions reduction efforts (ϵ, δ, γ) are larger), they have more enthusiasm to increase emissions reduction. As consumers become more environmentally conscious (i.e., the impact coefficients of product emissions reduction on product supply and demand, *α* and *φ*, are larger), the value chain participants prefer to make more emissions reduction investments. When the cost coefficients of emissions reduction investment (*μ*_*s*_, *μ*_*m*_, *μ*_*r*_) increase, or when the emissions reduction investment equipment is easily aged (i.e. the natural decay rate of product emissions reduction, *σ*, is large), the emissions reduction investment of participants will be hindered. In this case, the government could consider providing technology subsidies for value chain participants to promote emissions reduction.

Similarly, Zhang et al. [69] emphasized that increasing consumers’ awareness of low-carbon consumption had a significant positive effect on increasing companies’ emissions reduction investment. Moreover, their study suggested that increases in both the cost coefficients of emissions reduction investment and the decay rate discouraged manufacturers and retailers from such investments.

Expanding upon their research, we identify a positive correlation between the margin profit and participants’ emissions reduction efforts. This correlation also holds for the coefficients of emissions reduction efforts. In essence, our findings not only validate but also extend the insights provided by Zhang et al., offering a more detailed look at the financial motivations behind emissions reduction decisions.

Additionally, we compare and analyze the feedback equilibrium strategies and optimal profit obtained under the non-cooperative, cost-sharing and cooperative mechanisms, which yields Proposition 4.

#### Proposition 4

*The Pareto optimum of the agrifood value chain is achieved under the cooperative mechanism. The optimal emissions reduction efforts of the packer, producer and retailer are higher than the corresponding values in the non-cooperative and cost-sharing mechanisms, i.e.,* ${{{a_{m}^{c}}^{*}>a}_{m1}}^{*},{{{a_{m}^{c}}^{*}>a}_{m2}}^{*};\;{a_{s}^{c}}^{*}>{a_{s1}}^{*},{a_{s}^{c}}^{*}>{a_{s2}}^{*};\;{{{a_{r}^{c}}^{*}>a}_{r1}}^{*},{{{a_{r}^{c}}^{*}>a}_{r2}}^{*}$. The total profit of the agrifood value chain system is also higher than the corresponding values under the other two mechanisms, i.e., ${H_{w}^{c}}^{*}>{H_{1}}^{*},{H_{w}^{c}}^{*}>{H_{2}}^{*}$.

#### Proof of Proposition 4

The comparisons of game participants’ emissions reduction strategies and the agrifood value chain’s profit values under the non-cooperative, cost-sharing, and cooperative mechanisms are presented in Tables 1 and 2.

**Table 1:** Comparison of emissions reduction strategies.

Mechanisms	Participants	Comparative Results
Cooperative vs. Non-cooperative	Producer	${a_{s}^{c}}^{*}-{a_{s1}}^{*}=\frac{\varepsilon {(p}_{m}+p_{r})\varphi }{\mu_{s}(\rho +\sigma )}>0$
	Packer	${{{a_{m}^{c}}^{*}-a}_{m1}}^{*}=\frac{\delta {{(p}_{s}\alpha +p}_{r}\varphi )}{\mu_{m}(\rho +\sigma )}>0$
	Retailer	${{{a_{r}^{c}}^{*}-a}_{r1}}^{*}=\frac{\gamma {{(p}_{s}\alpha +p}_{m}\varphi )}{\mu_{r}(\rho +\sigma )}>0$
Cooperative vs. Cost-sharing	Producer	${{{a_{s}^{c}}^{*}-a}_{s2}}^{*}=\frac{\varepsilon {(p}_{s}\alpha +{2p}_{r}\varphi )}{2\mu_{s}(\rho +\sigma )}>0$
	Packer	${{{a_{m}^{c}}^{*}-a}_{m2}}^{*}=\frac{\delta {{(p}_{s}\alpha +p}_{r}\varphi )}{\mu_{m}(\rho +\sigma )}>0$
	Retailer	${{{a_{r}^{c}}^{*}-a}_{r2}}^{*}=\frac{\gamma {{(p}_{r}\varphi +2p}_{s}\alpha )}{2\mu_{r}(\rho +\sigma )}>0$

**Table 2:** Comparison of the profit of agrifood value chain.

Mechanisms	Comparative Results
Cooperative vs. Non-cooperative	${H_{w}^{c}}^{*}-{H_{1}}^{*}>0$
Cooperative vs. Cost-sharing	${H_{w}^{c}}^{*}-{H_{2}}^{*}>0$

in which ${H_{w}^{c}}^{*}-{H_{1}}^{*}=e^{-\rho t}(\frac{\varepsilon^{2}{\left(\alpha p_{s}+p_{r}\varphi \right)}^{2}}{2\rho \mu_{s}{\left(\rho +\sigma \right)}^{2}}+\frac{\delta^{2}\varphi^{2}{\left(p_{m}+p_{r}\right)}^{2}}{2\rho \mu_{m}{\left(\rho +\sigma \right)}^{2}}+\frac{\gamma^{2}{\left(\alpha p_{s}+p_{m}\varphi \right)}^{2}}{2\rho \mu_{r}{\left(\rho +\sigma \right)}^{2}})>0$ and ${H_{w}^{c}}^{*}-{H_{2}}^{*}=e^{-\rho t}(\frac{\varepsilon^{2}{\left(\alpha p_{s}+2p_{r}\varphi \right)}^{2}}{8\rho \mu_{s}{\left(\rho +\sigma \right)}^{2}}+\frac{\delta^{2}{\left(\alpha p_{s}+p_{r}\varphi \right)}^{2}}{2\rho \mu_{m}{\left(\rho +\sigma \right)}^{2}}+\frac{\gamma^{2}{\left(2\alpha p_{s}+p_{r}\varphi \right)}^{2}}{8\rho \mu_{r}{\left(\rho +\sigma \right)}^{2}})>0$. Thus, Proposition 4 is proven.

Proposition 4 clearly indicates that when compared to non-cooperative and cost-sharing mechanisms, the emissions reduction efforts by the producer, packer and retailer, as well as the profit of the agrifood value chain, are the highest under the cooperative mechanism. This observation confirms the findings of Chen et al. [70]. Their study emphasized the superiority of the cooperative mechanism over the non-cooperative one, providing a foundational understanding by focusing on emissions reduction under the two mechanisms. On this basis, we further investigate the effect of cost-sharing mechanism on participants’ dynamic decision-making, which has a more complex gaming process, marking a significant extension to their research. Moreover, we not only analyze the impact of the three mechanisms on emissions reduction, but also their effect on value chain profitability. This theoretical and following numerical examination of value chain profits further enhances our study. Our results suggest that upstream and downstream participants not only consider the maximization of long-term profit of the agrifood system but also follow the principle of the “economic-social-environmental” triple bottom line [71] when making dynamic decisions on vertical cooperation for emissions reduction.

Similar to our findings, many studies emphasized that within scenarios of vertical cooperation, environmental responsibility and profit aren’t mutually exclusive [72–76]. This underscores the potential of cooperative mechanisms to provide participants with a dual advantage: economic gain coupled with environmental benefits.

Our research results offer insights that can be adapted to other studies. Firstly, the evident advantage of the cooperative mechanism in both emissions reduction and profitability can be a focus for studies exploring collaboration dynamics, particularly in industries with a supply chain structure. Secondly, the results reveal emissions reduction decisions of the three participants under different collaborative mechanisms. Such insights can inform value chains with a similar three-stage structure, like the beef value chain that consists of the rancher, packer, and retailer. Lastly, by highlighting the relationship between marginal profits, emissions reduction efforts, associated coefficients, and emissions reduction, our findings offer guidance for studies in industries where the adoption of technology is critical, such as manufacturing, transportation, and energy.

## 4. Numerical analysis

To verify the validity of the models under the three mechanisms and analyze the impact of various parameters on product emissions reduction under the cooperative mechanism, we assign values to the relevant parameters and perform numerical analysis. The producer, packer, and retailer are assumed to obtain marginal profits *p*_*s*_ = 4, *p*_*m*_ = 6, and *p*_*r*_ = 5, respectively. The coefficients of their emissions reduction efforts on product emissions reduction are set to *ε* = 2, *δ* = 3, and *γ* = 3. The coefficients of the cost of emissions reduction are *μ*_*s*_ = 22, *μ*_*m*_ = 18, and *μ*_*r*_ = 12. The natural decay rate *σ* is 1, and the decay rate *ρ* is 0.8. The initial product supply and demand are set to *Q*_0_ = 14 and *D*_0_ = 12. The coefficients of the emissions reduction on market supply and demand are set to *α* = 2 and *φ* = 3, and the initial emissions reduction is assumed to be *q*(0) = 0.

### 4.1. Numerical results

Substituting the given parameter values into Proposition 1, Proposition 2 and Proposition 3, respectively, we can obtain the emissions reduction efforts of the producer, packer and retailer and the total profit of the agrifood value chain under different mechanisms as shown in Table 3.

**Table 3:** Numerical results under different mechanisms.

Mechanisms	*a*_s_*	*a*_m_*	*a*_r_*	Y*
Non-cooperative mechanism	0.4040	1.6667	2.0833	512.2736
Cost-sharing mechanism	1.1111	1.6667	3.5417	600.8492
Cooperative mechanism	2.0707	3.7963	5.6944	699.2914

Table 3 confirms Proposition 4, which states that the cooperative mechanism results in optimal emissions reduction efforts by the producer, packer, and retailer, compared to the non-cooperative and cost-sharing mechanisms. Specifically, the cooperative mechanism leads to total emissions reduction effort that is 2.78 times higher than that of the non-cooperative mechanism and 1.83 times higher than the cost-sharing mechanism. Additionally, the total profit of the agrifood value chain under the cooperative mechanism is also superior to the other two mechanisms, surpassing the non-cooperative mechanism and cost-sharing mechanism by 36.51% and 16.38%, respectively. In summary, long-term vertical collaboration in the agrifood value chain can incentivize all participants to invest in efforts to reduce emissions and lead to higher profit for the value chain.

The simulation experiment is conducted in Python, and the results presented in the figures reveal several important insights into the dynamics of emissions reduction efforts in the agrifood value chain. The resulting changes in product emissions reduction at different time intervals under the three mechanisms are displayed in Fig 2, in which $Y_{1}^{*},Y_{2}^{*}$, and $Y_{w}^{*}$ represent the profits of the whole agrifood value chain.

**Fig 4:**
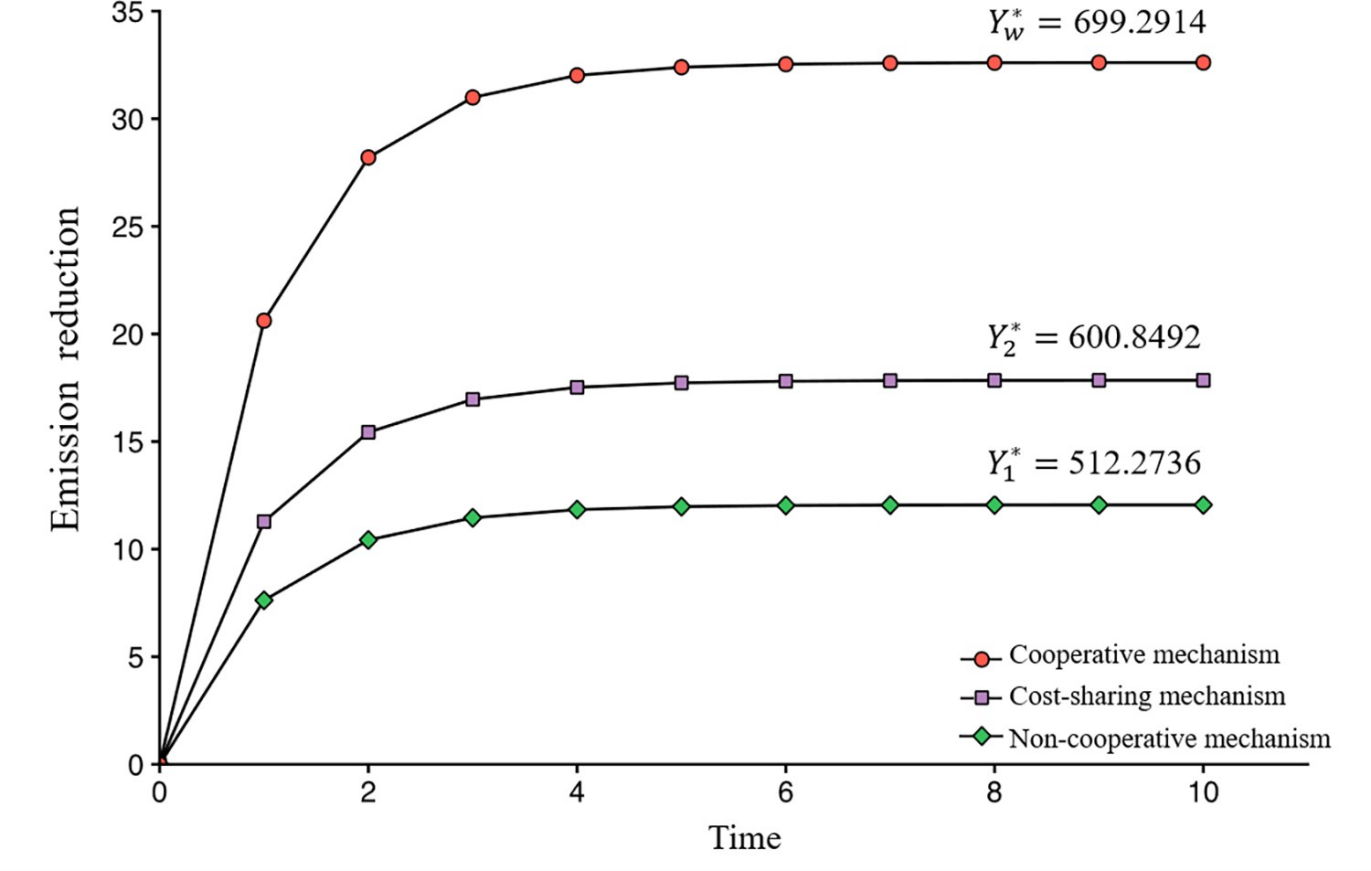
Curves of emissions reductions in different time intervals.

Fig 4 shows that compared to the non-cooperative and cost-sharing mechanisms, the cooperative mechanism leads to higher product emissions reductions and higher profits in agrifood value chain. This suggests that collaboration not only maximizes the profit of the agrifood value chain but also takes into account environmental factors, resulting in a significant reduction in emissions for the participants involved. Furthermore, it is worth noting that product emissions reduction gradually increases over time and eventually stabilizes regardless of the collaborative mechanism, indicating that external factors may influence the emissions reduction efforts, but eventually, the system returns to an equilibrium state.

### 4.2. Sensitivity analysis

In this section, we conduct a sensitivity analysis of the impact of four factors on emissions reductions: (1) the efforts made by the producer, packer, and retailer, (2) the emissions reduction costs, (3) the natural decay rate, (4) the impact of emissions reduction on agrifood products’ supply and demand.

**Fig 5:**
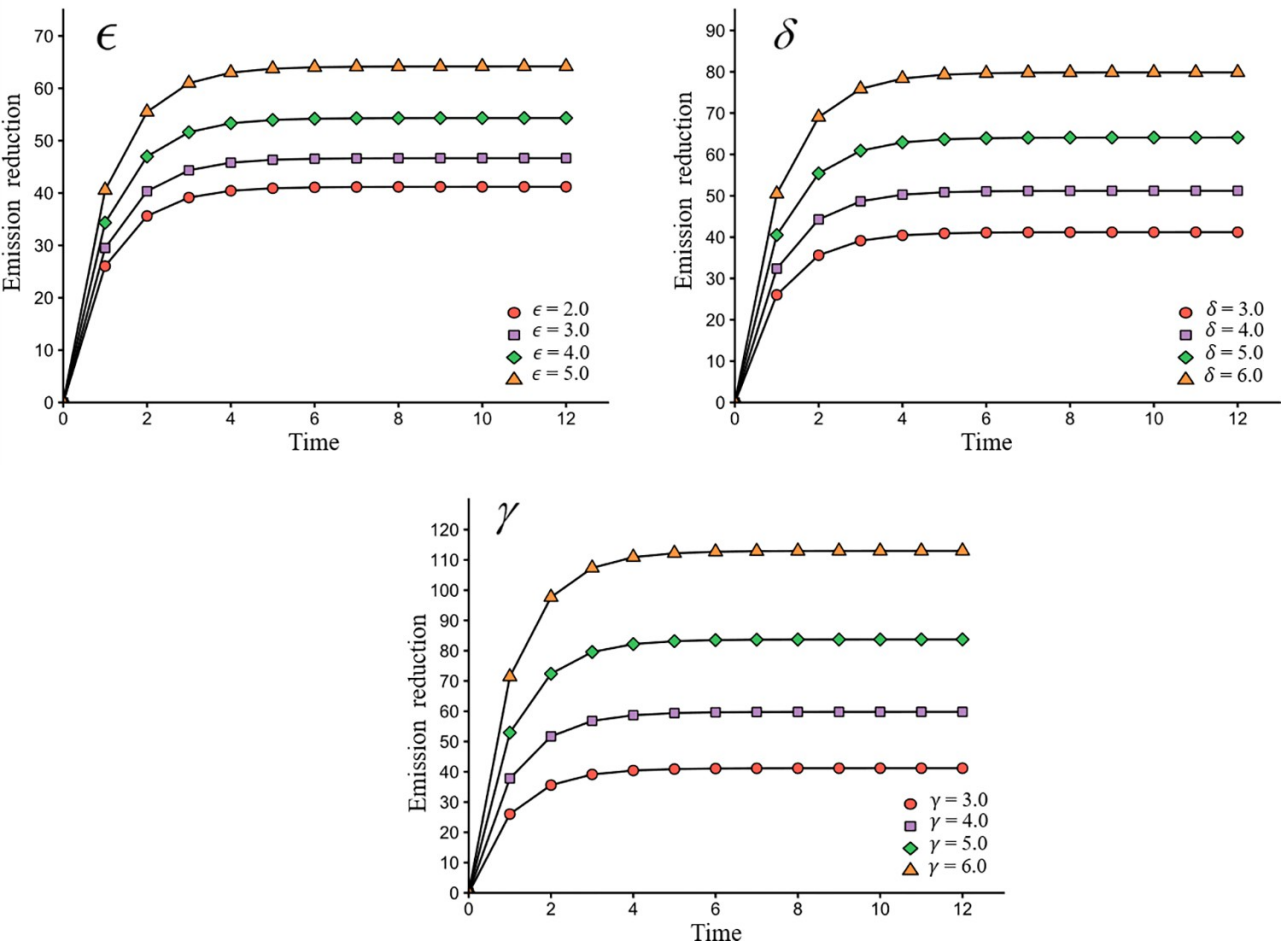
The impact of *ε*, *δ*, *γ* on emissions reduction.

Fig 5 displays the influence of efforts made by the producer, packer, and retailer on emissions reduction. This information is valuable for decision-makers to determine the optimal allocation of resources across the value chain to achieve emissions reduction goals.

We can observe that greater emissions reduction efforts lead to higher emissions reduction. In particular, the emissions reduction efforts of the retailer have a greater impact on product emissions reduction compared with the producer and packer. This suggests that low-emission promotion at the end of the agrifood value chain (i.e., to consumers) can contribute to a large emissions reduction in agrifood systems.

**Fig 6:**
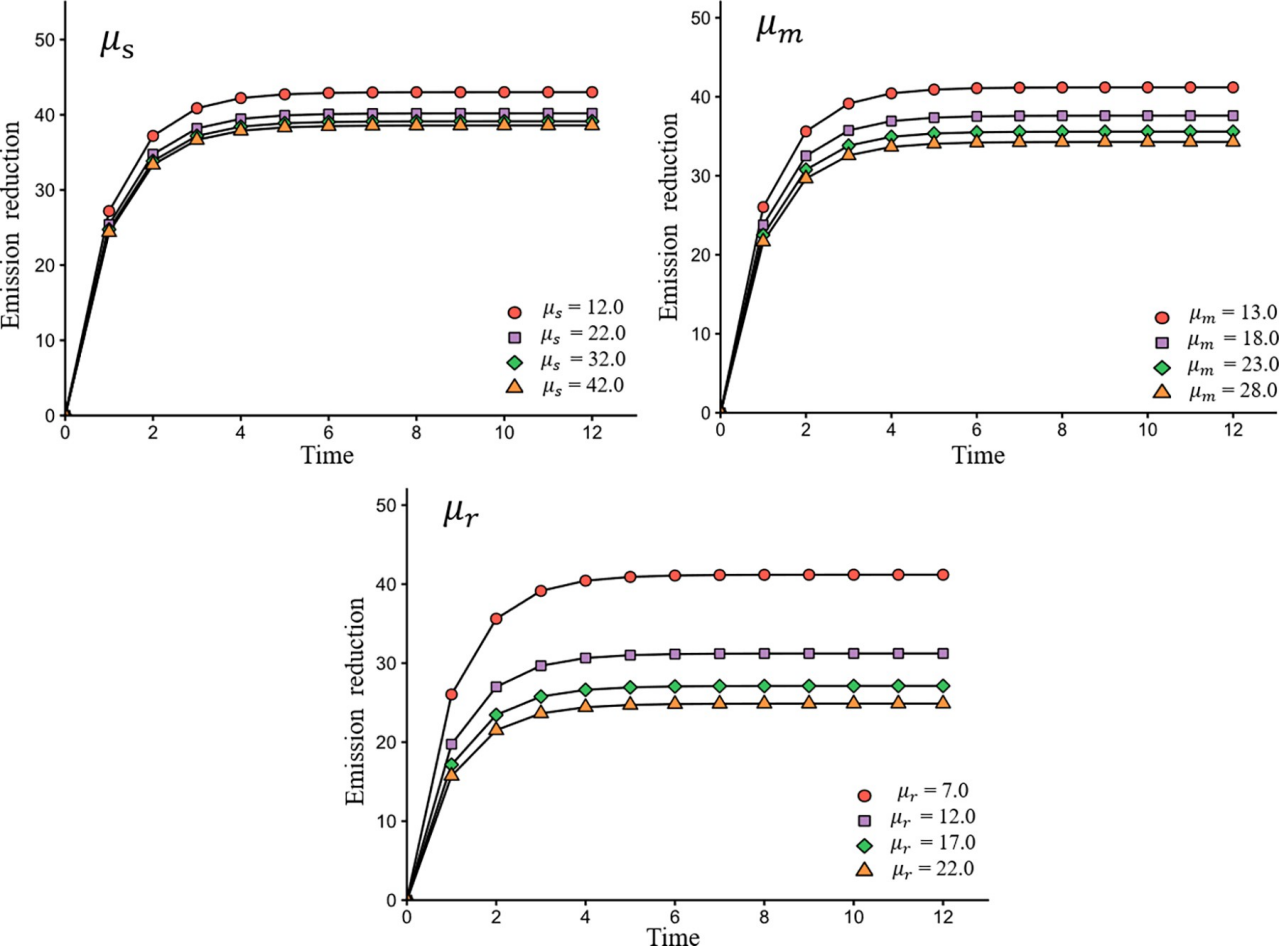
The impact of *μ*_*s*_, *μ*_*m*_, *μ*_*r*_ on emissions reduction.

Fig 6 provides insights into the impact of emissions reduction costs on emissions reduction efforts. This figure allows decision-makers to understand better the trade-offs between cost and emissions reduction efforts in the agrifood value chain.

From Fig 6, it is clear that the higher the emissions reduction cost borne by the agrifood value chain participants, the lower the emissions reduction will be. Reasonably, excessive emissions reduction costs are likely to discourage participants from paying more to reduce emissions. Hence, in the actual operation of the agrifood value chain, reducing the cost of emissions reduction through technological innovation is also an important method in meeting emissions reduction targets.

**Fig 7:**
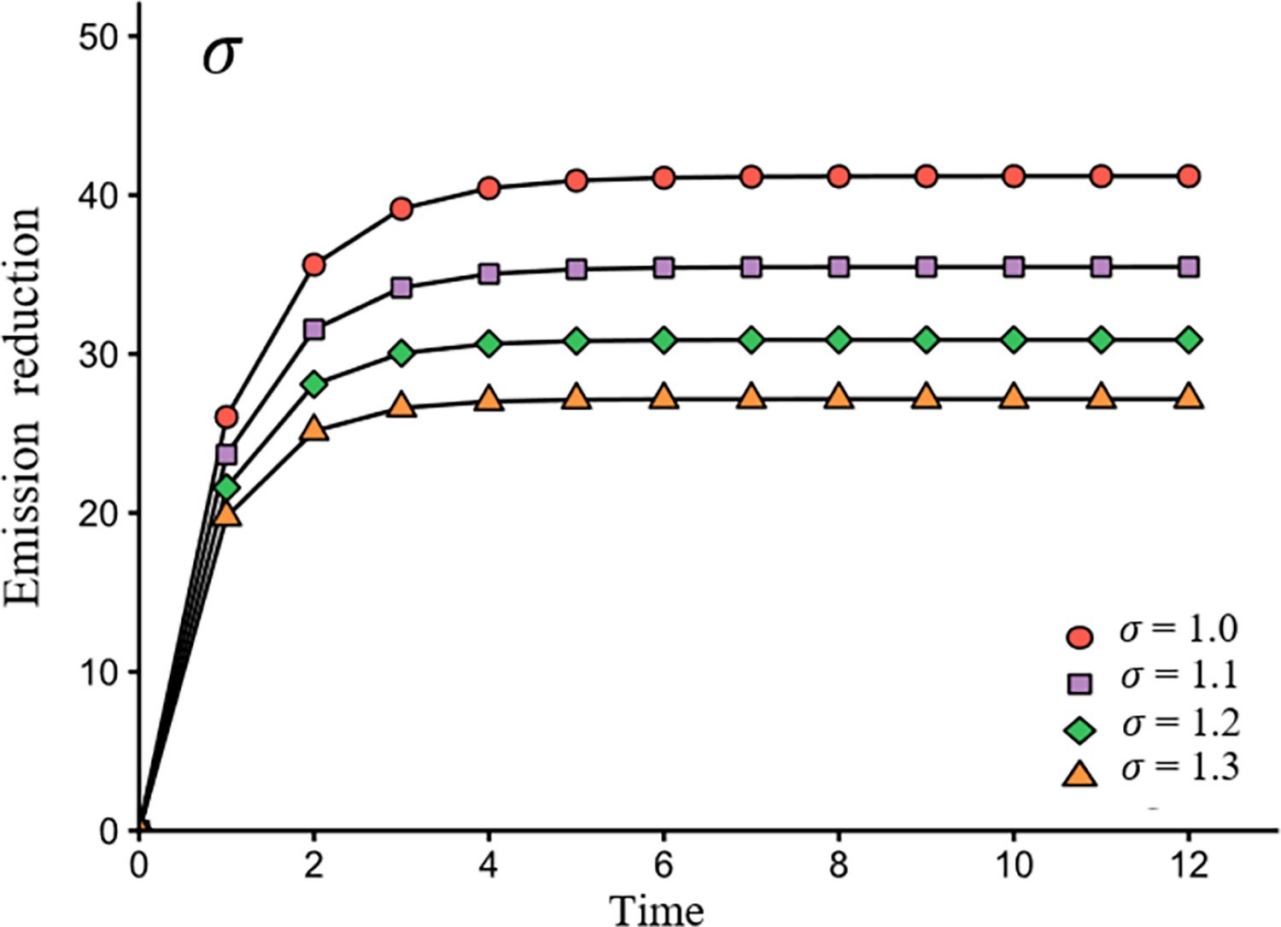
The impact of *σ* on emissions reduction.

Fig 7 shows the impact of the natural decay rate on emissions reduction. This figure can help decision-makers understand the role of the natural decay rate in achieving emissions reduction goals and can assist them in developing strategies to maximize emissions reduction.

In Fig 7, it’s evident that as the natural decay rate increases, the product emissions reduction decreases. This indicates that higher natural decay rates lead to increased costs for companies to operate emissions reduction equipment, making it even more challenging for them to achieve long-term emissions reduction goals.

**Fig 8:**
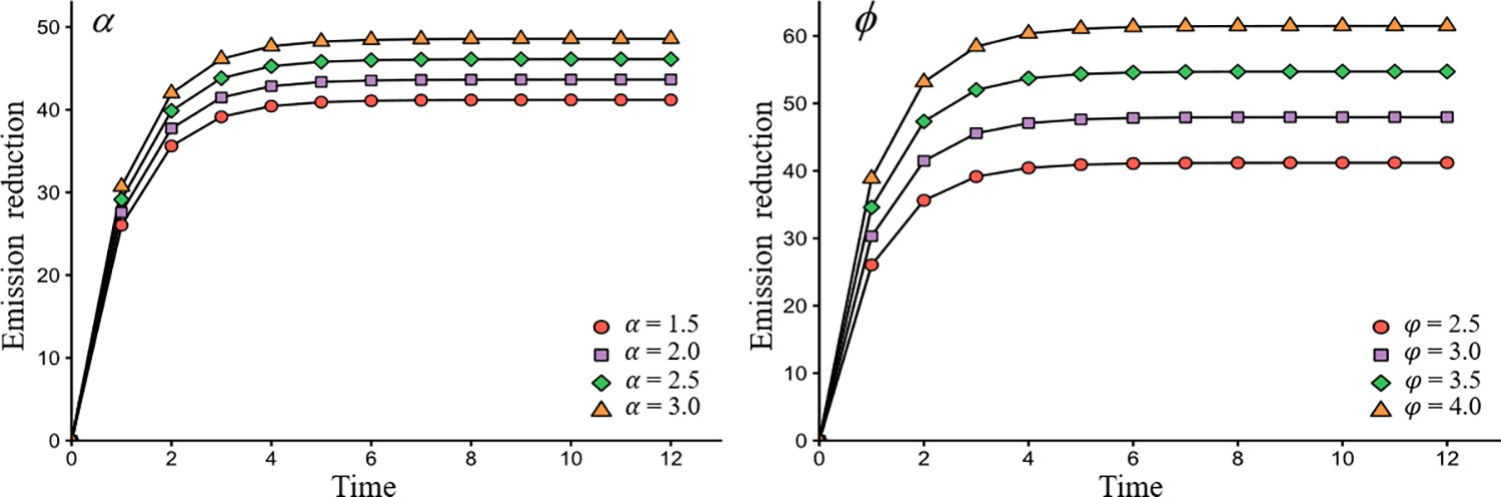
The impact of *α*, *φ* on emissions reduction.

In Fig 8, *α* and *φ* represent the impact of emissions reduction on agrifood products’ supply and demand. And Fig 8 depicts the impact of *α* and *φ* on emissions reduction, providing insights into the potential economic implications of emissions reduction efforts, such as changes in supply and demand in the market.

Fig 8 shows that when the emissions reduction has a greater impact on agricultural product supply and demand, the overall emissions reduction of the agrifood value chain is higher, indicating that consumers’ low-emission preferences have a large impact on the supply and demand for low-emission agrifood products. Thus, the producers, packers and retailers should firmly grasp the low-emission preferences of consumers and put more effort into reducing emissions to obtain higher profits.

From the sensitivity analysis figures, emissions reduction efforts show the most significant and positive impact on emissions reduction effectiveness, compared to the other three factors. This finding is consistent with the reality that greater efforts in emissions reduction yield more significant results. Similarly, a positive market response in terms of supply and demand to emissions reduction further boosts the decrease in emissions. In contrast, as emissions reduction costs and the natural decay rate increase, the level of emissions reduction gradually decreases. The results illustrate that emissions reduction is a dynamic, long-term, and complex process, and that it is important for value chain participants to consider the changes in each factor comprehensively in order to make optimal time-varying emissions reduction decisions.

## 5. Conclusion

Emissions reduction in the agrifood systems is particularly challenging because the benefits and costs of emissions reduction are borne by different agrifood value chain participants. Emissions reduction collaboration among value chain participants could potentially correct those misalignments and maximize the benefits of the entire value chain. In this paper, we develop a dynamic differential game model to examine participants’ long-term emissions reduction strategies in a three-stage agrifood value chain. We use Hamilton-Jacobi-Bellman (HJB) equation to derive the value chain participants’ long-term Nash equilibrium emissions reduction strategies under non-cooperative, cost-sharing, and cooperative mechanisms.

Our results show that collaboration among value chain participants leads to higher emissions reduction efforts and higher profits for the entire value chain. Specifically, the cooperative mechanism resulted in the highest emissions reduction effort and the highest profit for the entire value chain. Under all three collaborative mechanisms, value chain participants are willing to put forth more emissions reduction efforts when those efforts can be easily translated into emissions reduction in the final product and when consumers become more environmentally conscious. We also find that reducing the cost of emissions reduction through technological innovation is an important method of meeting emissions reduction targets.

Based on these findings, several practical and managerial implications can be inferred. Policymakers can design supportive policies or strategies that promote and incentivize collaboration among value chain participants, such as offering subsidies for emissions reduction. Furthermore, industry stakeholders can adapt their approaches to achieve optimal emissions reduction, while considering factors like marginal profits, demand-supply dynamics, and consumer preferences. Given that the cooperative approach yields the highest profits for the entire value chain, decision-makers in the agrifood industry should consider implementing collaboration not only to achieve emissions reduction goals but also to enhance profitability. Moreover, we emphasize the importance of technological innovation in achieving emissions reduction targets. Innovations that reduce the cost of emissions reduction efforts can be a powerful tool for enterprises to meet sustainability goals while maintaining profitability. Future research can investigate the effectiveness and distributional effects of different policies and regulations in facilitating collaborations among value chain participants.

Nonetheless, there are several potential limitations and research directions for further investigation. Firstly, we would like to point out that our results are specific to the three-stage agrifood value chain, where the packer is the leader in the value chain, and the producer and the retailer are followers. The packer is incentivized to encourage emissions reduction collaborations among value chain participants. In many agrifood industries where the final product is sold under the packer’s brand (e.g., Danone’s yogurt products, General Mills’s cereal products), this setup is appropriate. However, there are also cases where the retailer (e.g., Walmart) or food service distributor (e.g., McDonald’s) is the leader of the value chain, and the producer and the packer are followers. Our results may not hold for those types of agrifood value chains. Further research is needed to confirm whether the cooperative mechanism can achieve the highest profit and largest emissions reduction across various types of agrifood value chains.

Secondly, we assume that the marginal profits of the producer, packer and retailer are time-invariant parameters. We also assume that the demand and supply of low-emission agrifood products are linear, and emissions reduction costs are quadratic functions of each value chain participant’s emissions reduction effort. We are able to derive an analytical solution of the value chain participants’ emissions reduction strategies and their resulting profits. Nonetheless, we admit that the actual agrifood value chain is much more complex and is increasingly so. To fully reflect the complexity and power dynamics of real-world agrifood value chains, future research can expand our model by introducing more value chain participants, assuming different value chain structures, and incorporating more complicated consumer demand models, among other extensions.

## Supporting information

S1 Table The descriptions of parameters. (DOCX)

S1 File Proofs. (DOCX)
