# Supplementary material for: Emissions reduction strategy in a three-stage agrifood value chain: A dynamic differential game approach
Source: PLoS One. 2023 Nov 17;18(11):e0294472. doi: 10.1371/journal.pone.0294472 (PMC10656033; doi:10.1371/journal.pone.0294472)
Supplement: S1 Table — (DOCX) [file pone.0294472.s001.docx]

**Supporting information**

**Table 1. The descriptions of parameters**

| **Parameter** | **Description** |
| --- | --- |
| $p_{s}$ | marginal profit of producer $s$ |
| $p_{m}$ | marginal profit of packer $m$ |
| $p_{r}$ | marginal profit of retailer $r$ |
| $a_{s}(t)$ | effort of producer $s$ to reduce carbon emissions at time $t$ |
| $a_{m}\left( t \right)$ | effort of packer $m$ to reduce carbon emissions at time $t$ |
| $a_{r}(t)$ | effort of retailer $r$ to promote low-emission products at time $t$ |
| $q(t)$ | emissions reduction of the product at time $t$, $q\left( 0 \right)\geq0$ |
| $\beta(t)$ | subsidy ratio of the packer $m$ to the producer $s$ at time $t$, $0\leq\beta\left( t \right)\leq1$ |
| $\theta(t)$ | subsidy ratio of the packer $m$ to the retailer $r$ at time $t$, $0\leq\theta\left( t \right)\leq1$ |
| $H_{s}$ | present profit function of producer $s$ (infinite time horizon) |
| $H_{m}$ | present profit function of packer $m$ (infinite time horizon) |
| $H_{r}$ | present profit function of retailer $r$ (infinite time horizon) |
| $H_{w}^{c}$ | present profit function of the agrifood value chain (infinite time horizon) |
| $Y_{s}$ | profit function of producer $s$ |
| $Y_{m}$ | profit function of packer $m$ |
| $Y_{r}$ | profit function of retailer $r$ |
| $Y_{w}$ | profit function of the agrifood value chain |
| $D(t)$ | demand of product, initial demand $D_{0}\geq0$ |
| $Q(t)$ | supply of product, initial supply $Q_{0}\geq0$ |
