# Supplementary material for: Emissions reduction strategy in a three-stage agrifood value chain: A dynamic differential game approach
Source: PLoS One. 2023 Nov 17;18(11):e0294472. doi: 10.1371/journal.pone.0294472 (PMC10656033; doi:10.1371/journal.pone.0294472)
Supplement: S1 File — (DOCX) [file pone.0294472.s002.docx]

**Supporting information**

**Proof of Proposition 1**

The optimal value functions of the total present profit of producer, packer and retailer after time $t$ are as follows:

| $H_{s1}^{*}\left( q \right)=\max_{a_{s1}}\int_{t}^{\infty} e^{-\rho\tau}\left\{ p_{s}Q\left( q\left( \tau\right), \tau\right)-\frac{\mu_{s}}{2}\left[ a_{s1}\left( \tau\right) \right]^{2} \right\}d\tau$ | (A.1) |
| --- | --- |
| $H_{m1}^{*}\left( q \right)=\max_{a_{m1}}\int_{t}^{\infty} e^{-\rho\tau}\left\{ p_{m}D\left( q\left( \tau\right), \tau\right)-\frac{\mu_{m}}{2}\left[ a_{m1}\left( \tau\right) \right]^{2} \right\}d\tau$ | (A.2) |
| $H_{r1}^{*}\left( q \right)=\max_{a_{r1}}\int_{0}^{\infty} e^{-\rho\tau}\left\{ p_{r}D\left( q\left( \tau\right), \tau\right)-\frac{\mu_{r}}{2}\left[ a_{r1}\left( \tau\right) \right]^{2} \right\}d\tau$ | (A.3) |

Let the optimal value functions of the total profit of producer, packer and retailer after time $t$ be

| $Y_{s1}\left( q \right)= \max_{a_{s1}}\int_{t}^{\infty} e^{-\rho(\tau-t)}\left\{ p_{s}Q\left( q\left( \tau\right), \tau\right)-\frac{\mu_{s}}{2}\left[ a_{s1}\left( \tau\right) \right]^{2} \right\}d\tau$ | (A.4) |
| --- | --- |
| $Y_{m1}\left( q \right)= \max_{a_{m1}}\int_{t}^{\infty} e^{-\rho(\tau-t)}\left\{ p_{m}D\left( q\left( \tau\right), \tau\right)-\frac{\mu_{m}}{2}\left[ a_{m1}\left( \tau\right) \right]^{2} \right\}d\tau$ | (A.5) |
| $Y_{r1}\left( q \right)= \max_{a_{r1}}\int_{t}^{\infty} e^{-\rho(\tau-t)}\left\{ p_{r}D\left( q\left( \tau\right), \tau\right)-\frac{\mu_{r}}{2}\left[ a_{r1}\left( \tau\right) \right]^{2} \right\}d\tau$ | (A.6) |

Then the optimal value functions of the total present profit of producer, packer and retailer after time $t$ can be expressed as

| $H_{s1}^{*}\left( q \right)= e^{-\rho t}Y_{s1}\left( q \right)$ | (A.7) |
| --- | --- |
| $H_{m1}^{*}\left( q \right)= e^{-\rho t}Y_{m1}\left( q \right)$ | (A.8) |
| $H_{r1}^{*}\left( q \right)= e^{-\rho t}Y_{r1}\left( q \right)$ | (A.9) |

Then $Y_{s1}\left( q \right)$, $Y_{m1}\left( q \right){, Y}_{r1}\left( q \right)$ must satisfy the following HJB equation for all $q \geq0$, respectively.

| $\rho Y_{s1}\left( q \right)=\max_{a_{s1}} [p_{s}\left( Q_{0}+\alpha q \right)-\frac{\mu_{s}}{2}{a_{s1}}^{2}+{Y_{s1}\left( q \right)}^{'} (\varepsilon a_{s1}+\delta a_{m1}+\gamma a_{r1}-\sigma q)]$ | (A.10) |
| --- | --- |
| $\rho Y_{m1}\left( q \right)=\max_{a_{m1}} \left[ p_{m}\left( D_{0}+\varphi q \right)-\frac{\mu_{m}}{2}{a_{m1}}^{2}+{Y_{m1}\left( q \right)}^{'} \left( \varepsilon a_{s1}+\delta a_{m1}+\gamma a_{r1}-\sigma q \right) \right]$ | (A.11) |
| $\rho Y_{r1}\left( q \right)=\max_{a_{r1}} [p_{r}\left( D_{0}+\varphi q \right)-\frac{\mu_{r}}{2}{a_{r1}}^{2}+{Y_{r1}\left( q \right)}^{'} (\varepsilon a_{s1}+\delta a_{m1}+\gamma a_{r1}-\sigma q)]$ | (A.12) |

Obviously, Eq. (A.10), (A.11) and (A.12) are concave functions with respect to $a_{s1}$, $a_{m1}$ and $a_{r1}$ respectively, which can be solved by the first-order condition.

| $a_{s1}=\frac{\varepsilon{Y_{s1}\left( q \right)}^{'}}{\mu_{s}}$ | (A.13) |
| --- | --- |
| $a_{m1}=\frac{\delta{Y_{m1}\left( q \right)}^{'}}{\mu_{m}}$ | (A.14) |
| $a_{r1}=\frac{\gamma{Y_{r1}\left( q \right)}^{'}}{\mu_{r}}$ | (A.15) |

Substituting Eq. (A.13), (A.14), (A.15) into Eq. (A.10), (A.11) and (A.12).

| $\rho Y_{s1}\left( q \right)= \left( p_{s}\alpha-\sigma{Y_{s1}\left( q \right)}^{'} \right)q+p_{s}Q_{0}+\frac{\varepsilon^{2}{{Y_{s1}\left( q \right)}^{'}}^{2}}{{2\mu}_{s}}+\frac{\delta^{2}{Y_{s1}\left( q \right)}^{'}{Y_{m1}\left( q \right)}^{'}}{\mu_{m}}+\frac{\gamma^{2}{Y_{s1}\left( q \right)}^{'}{Y_{r1}\left( q \right)}^{'}}{\mu_{r}}$ | (A.16) |
| --- | --- |
| $\rho Y_{m1}\left( q \right)= \left( p_{m}\varphi-\sigma{Y_{m1}\left( q \right)}^{'} \right)q+p_{m}D_{0}+\frac{\varepsilon^{2}{Y_{m1}\left( q \right)}^{'}{Y_{s1}\left( q \right)}^{'}}{\mu_{s}}+\frac{\delta^{2}{{Y_{m1}\left( q \right)}^{'}}^{2}}{{2\mu}_{m}}+\frac{\gamma^{2}{Y_{m1}\left( q \right)}^{'}{Y_{r1}\left( q \right)}^{'}}{\mu_{r}}$ | (A.17) |
| $\rho Y_{r1}\left( q \right)= \left( p_{r}\varphi-\sigma{Y_{r1}\left( q \right)}^{'} \right)q+p_{r}D_{0}+\frac{\varepsilon^{2}{Y_{r1}\left( q \right)}^{'}{Y_{s1}\left( q \right)}^{'}}{\mu_{s}}+\frac{\delta^{2}{{{Y_{r1}\left( q \right)}^{'}Y}_{m1}\left( q \right)}^{'}}{\mu_{m}}+\frac{\gamma^{2}{{Y_{r1}\left( q \right)}^{'}}^{2}}{2\mu_{r}}$ | (A.18) |

Noting that Eq. (A.16), (A.17) and (A.18) are all first-order differential equations, it is presumed that the linear function on $q$ is the solution of the HJB equation.

| $Y_{s1}\left( q \right)= k_{1}q+b_{1}; Y_{m1}\left( q \right)= k_{2}q+b_{2}; Y_{r1}\left( q \right)= k_{3}q+b_{3}$ | (A.19) |
| --- | --- |

where $k_{1}$, $b_{1}$, $k_{2}$, $b_{2}$, $k_{3}$, $b_{3}$ are constants. The first order derivatives with respect to $q$ are obtained separately from Eq. (A.19).

| ${Y_{s1}\left( q \right)}^{'}= k_{1}; {Y_{m1}\left( q \right)}^{'}= k_{2}; {Y_{r1}\left( q \right)}^{'}= k_{3}$ | (A.20) |
| --- | --- |

Substituting Eq. (A.19) and (A.20) into Eq. (A.16), (A.17) and (A.18), respectively.

| $\rho(k_{1}q+b_{1})= \left( p_{s}\alpha-\sigma k_{1} \right)q+p_{s}Q_{0}+\frac{\varepsilon^{2}{k_{1}}^{2}}{{2\mu}_{s}}+\frac{\delta^{2}k_{1}k_{2}}{\mu_{m}}+\frac{\gamma^{2}k_{1}k_{3}}{\mu_{r}}$ | (A.21) |
| --- | --- |
| $\rho\left( k_{2}q+b_{2} \right)= \left( p_{m}\varphi-\sigma k_{2} \right)q+p_{m}D_{0}+\frac{\varepsilon^{2}k_{2}k_{1}}{\mu_{s}}+\frac{\delta^{2}{k_{2}}^{2}}{{2\mu}_{m}}+\frac{\gamma^{2}k_{2}k_{3}}{\mu_{r}}$ | (A.22) |
| $\rho(k_{3}q+b_{3})= \left( p_{r}\varphi-\sigma k_{3} \right)q+p_{r}D_{0}+\frac{\varepsilon^{2}k_{3}k_{1}}{\mu_{s}}+\frac{\delta^{2}k_{3}k_{2}}{\mu_{m}}+\frac{\gamma^{2}{k_{3}}^{2}}{2\mu_{r}}$ | (A.23) |

Comparing the coefficients of similar terms on both sides of Eq. (A.21), (A.22) and (A.23), we can obtain the equations system for $k_{1}$, $b_{1}$, $k_{2}$, $b_{2}$, $k_{3}$, $b_{3}$.

| $\rho k_{1}= p_{s}\alpha-\sigma k_{1}$ | (A.24) |
| --- | --- |
| $\rho b_{1}= p_{s}Q_{0}+\frac{\varepsilon^{2}{k_{1}}^{2}}{{2\mu}_{s}}+\frac{\delta^{2}k_{1}k_{2}}{\mu_{m}}+\frac{\gamma^{2}k_{1}k_{3}}{\mu_{r}}$ |  |
| $\rho k_{2}= p_{m}\varphi-\sigma k_{2}$ |  |
| $\rho b_{2}= p_{m}D_{0}+\frac{\varepsilon^{2}k_{2}k_{1}}{\mu_{s}}+\frac{\delta^{2}{k_{2}}^{2}}{{2\mu}_{m}}+\frac{\gamma^{2}k_{2}k_{3}}{\mu_{r}}$ |  |
| $\rho k_{3}= p_{r}\varphi-\sigma k_{3}$ |  |
| $\rho b_{3}= p_{r}D_{0}+\frac{\varepsilon^{2}k_{3}k_{1}}{\mu_{s}}+\frac{\delta^{2}k_{3}k_{2}}{\mu_{m}}+\frac{\gamma^{2}{k_{3}}^{2}}{2\mu_{r}}$ |  |

Solving the equations system (A.24), we can get the results of $k_{1}$, $b_{1}$, $k_{2}$, $b_{2}$, $k_{3}$, $b_{3}$.

| $k_{1}= \frac{p_{s}\alpha}{\rho+\sigma}$ | (A.25) |
| --- | --- |
| $b_{1}= \frac{p_{s}Q_{0}}{\rho}+\frac{\varepsilon^{2}{k_{1}}^{2}}{{2\rho\mu}_{s}}+\frac{\delta^{2}k_{1}k_{2}}{{\rho\mu}_{m}}+\frac{\gamma^{2}k_{1}k_{3}}{\rho\mu_{r}}$ |  |
| $k_{2}= \frac{p_{m}\varphi}{\rho+\sigma}$ |  |
| $b_{2}= \frac{p_{m}D_{0}}{\rho}+\frac{\varepsilon^{2}k_{2}k_{1}}{\rho\mu_{s}}+\frac{\delta^{2}{k_{2}}^{2}}{{2\rho\mu}_{m}}+\frac{\gamma^{2}k_{2}k_{3}}{\rho\mu_{r}}$ |  |
| $k_{3}= \frac{p_{r}\varphi}{\rho+\sigma}$ |  |
| $b_{3}=\frac{p_{r}D_{0}}{\rho}+\frac{\varepsilon^{2}k_{3}k_{1}}{\rho\mu_{s}}+\frac{\delta^{2}k_{3}k_{2}}{{\rho\mu}_{m}}+\frac{\gamma^{2}{k_{3}}^{2}}{2\rho\mu_{r}}$ |  |

Substituting equations system (A.25) into Eq. (A.20).

| ${Y_{s1}\left( q \right)}^{'}= \frac{p_{s}\alpha}{\rho+\sigma}; {Y_{m1}\left( q \right)}^{'}= \frac{p_{m}\varphi}{\rho+\sigma}; {Y_{r1}\left( q \right)}^{'}=\frac{p_{r}\varphi}{\rho+\sigma}$ | (A.26) |
| --- | --- |

Substituting Eq. (A.26) into Eq. (A.13), (A.14) and (A.15).

| ${a_{s1}}^{*}=\frac{\varepsilon p_{s}\alpha}{\mu_{s}(\rho+\sigma)}$ | (A.27) |
| --- | --- |
| ${a_{m1}}^{*}=\frac{\delta p_{m}\varphi}{\mu_{m}(\rho+\sigma)}$ | (A.28) |
| ${a_{r1}}^{*}=\frac{\gamma p_{r}\varphi}{\mu_{r}(\rho+\sigma)}$ | (A.29) |

Substituting equations system (A.25) into Eq. (A.19), we can derive the optimal value functions of the total profit of the producer, packer and retailer.

| ${Y_{s1}\left( q \right)}^{*}= \frac{p_{s}\alpha}{\rho+\sigma}q+ \frac{p_{s}Q_{0}}{\rho}+\frac{\varepsilon^{2}{p_{s}}^{2}\alpha^{2}}{{2\rho\mu}_{s}{(\rho+\sigma)}^{2}}+\frac{\delta^{2}\alpha\varphi p_{s}p_{m}}{{\rho\mu}_{m}{(\rho+\sigma)}^{2}}+\frac{\gamma^{2}\alpha\varphi p_{s}p_{r}}{\rho\mu_{r}{(\rho+\sigma)}^{2}}$ | (A.30) |
| --- | --- |
| ${Y_{m1}\left( q \right)}^{*}= \frac{p_{m}\varphi}{\rho+\sigma}q+ \frac{p_{m}D_{0}}{\rho}+\frac{\varepsilon^{2}\varphi{\alpha p}_{m}p_{s}}{\rho\mu_{s}{(\rho+\sigma)}^{2}}+\frac{\delta^{2}\varphi^{2}{p_{m}}^{2}}{{2\rho\mu}_{m}{(\rho+\sigma)}^{2}}+\frac{\gamma^{2}{\varphi^{2}p}_{m}p_{r}}{\rho\mu_{r}{(\rho+\sigma)}^{2}}$ | (A.31) |
| ${Y_{r1}\left( q \right)}^{*}=\frac{p_{r}\varphi}{\rho+\sigma}q+\frac{p_{r}D_{0}}{\rho}+\frac{\varepsilon^{2}{\alpha\varphi p}_{r}p_{s}}{\rho\mu_{s}{(\rho+\sigma)}^{2}}+\frac{\delta^{2}\varphi^{2}p_{r}p_{m}}{{\rho\mu}_{m}{(\rho+\sigma)}^{2}}+\frac{\gamma^{2}{{p_{r}}^{2}\varphi}^{2}}{2\rho\mu_{r}{(\rho+\sigma)}^{2}}$ | (A.32) |

Substituting Eq. (A.30), (A.31) and (A.32) into Eq. (A.7), (A.8), (A.9), we can obtain the optimal value functions of the total present profit of the producer, packer and retailer.

| $H_{s1}^{*}\left( q \right)= e^{-\rho t}{Y_{s1}\left( q \right)}^{*}$ | (A.33) |
| --- | --- |
| $H_{m1}^{*}\left( q \right)= e^{-\rho t}{Y_{m1}\left( q \right)}^{*}$ | (A.34) |
| $H_{r1}^{*}\left( q \right)= e^{-\rho t}{Y_{r1}\left( q \right)}^{*}$ | (A.35) |

Then, the trajectory of the product emissions reduction can be solved by substituting Eq. (A.27), (A.27) and (A.27) into the state equation Eq. (1).

| $q\left( t \right)= z-(z-q_{0})e^{-\sigma t}$ | (A.36) |
| --- | --- |

where $z= \frac{\varepsilon^{2}p_{s}\alpha}{\sigma\mu_{s}(\rho+\sigma)}+\frac{\delta^{2}p_{m}\varphi}{\sigma\mu_{m}(\rho+\sigma)}+\frac{\gamma^{2}p_{r}\varphi}{\sigma\mu_{r}(\rho+\sigma)}$. Thus, Proposition 1 is proven.

Finally, let ${H_{1}}^{*}(q)$ denote the present profit of the whole agrifood value chain.

| ${H_{1}}^{*}(q)=H_{s1}^{*}\left( q \right)+H_{m1}^{*}\left( q \right)+H_{r1}^{*}\left( q \right)$ | (A.37) |
| --- | --- |

**Proof of Proposition 2**

To obtain feedback Stackelberg equilibrium strategy for this game, we use the backward induction method. Firstly, the producer and retailer, as followers, decide the optimal level of emissions reduction effort by taking the packer’s emissions reduction effort $a_{m2}$ and cost-sharing ratio $\beta(t)$, $\theta(t)$ as given parameters. This translates into a unilateral optimal control problem for producer and retailer. From Eq. (11) and (12), we denote the optimal value functions of the total present profit of the producer and retailer after time $t$ as

| $H_{s2}^{*}\left( q \right)=\max_{a_{s2}}\int_{t}^{\infty} e^{-\rho\tau}\left\{ p_{s}Q\left( q\left( \tau\right), \tau\right)-\left( 1-\beta\right)\frac{\mu_{s}}{2}\left[ a_{s2}\left( \tau\right) \right]^{2} \right\}d\tau$ | (A.38) |
| --- | --- |
| $H_{r2}^{*}\left( q \right)=\max_{a_{r2}}\int_{0}^{\infty} e^{-\rho\tau}\left\{ p_{r}D\left( q\left( \tau\right), \tau\right)-\left( 1-\theta\right)\frac{\mu_{r}}{2}\left[ a_{r2}\left( \tau\right) \right]^{2} \right\}d\tau$ | (A.39) |

Let the optimal value functions of the total profit of the producer and retailer after time $t$ be

| $Y_{s2}\left( q \right)= \max_{a_{s2}}\int_{t}^{\infty} e^{-\rho(\tau-t)}\left\{ p_{s}Q\left( q\left( \tau\right), \tau\right)-\left( 1-\beta\right)\frac{\mu_{s}}{2}\left[ a_{s2}\left( \tau\right) \right]^{2} \right\}d\tau$ | (A.40) |
| --- | --- |
| $Y_{r2}\left( q \right)= \max_{a_{r2}}\int_{t}^{\infty} e^{-\rho(\tau-t)}\left\{ p_{r}D\left( q\left( \tau\right), \tau\right)-\left( 1-\theta\right)\frac{\mu_{r}}{2}\left[ a_{r2}\left( \tau\right) \right]^{2} \right\}d\tau$ | (A.41) |

Then the optimal value functions of the total present profit of the producer and retailer after time $t$ can be expressed as

| $H_{s2}^{*}\left( q \right)= e^{-\rho t}Y_{s2}\left( q \right)$ | (A.42) |
| --- | --- |
| $H_{r2}^{*}\left( q \right)= e^{-\rho t}Y_{r2}\left( q \right)$ | (A.43) |

Then $Y_{s2}\left( q \right)$ and $Y_{r2}\left( q \right)$ must satisfy the following HJB equation for all $q \geq0$, respectively.

| $\rho Y_{s2}\left( q \right)=\max_{a_{s2}} [p_{s}\left( Q_{0}+\alpha q \right)-\left( 1-\beta\right)\frac{\mu_{s}}{2}{a_{s2}}^{2}+{Y_{s2}\left( q \right)}^{'} (\varepsilon a_{s2}+\delta a_{m2}+\gamma a_{r2}-\sigma q)]$ | (A.44) |
| --- | --- |
| $\rho Y_{r2}\left( q \right)=\max_{a_{r2}} [p_{r}\left( D_{0}+\varphi q \right)-\left( 1-\theta\right)\frac{\mu_{r}}{2}{a_{r2}}^{2}+{Y_{r2}\left( q \right)}^{'} (\varepsilon a_{s2}+\delta a_{m2}+\gamma a_{r2}-\sigma q)]$ | (A.45) |

Obviously, Eq. (A.44) and (A.45) are both concave functions with respect to $a_{s2}$ and $a_{r2}$ respectively, which can be solved by the first order condition.

| $a_{s2}=\frac{\varepsilon{Y_{s2}\left( q \right)}^{'}}{\left( 1-\beta\right)\mu_{s}}$ | (A.46) |
| --- | --- |
| $a_{r2}=\frac{\gamma{Y_{r2}\left( q \right)}^{'}}{\left( 1-\theta\right)\mu_{r}}$ | (A.47) |

Considering the producer and retailer will adopt their own optimal strategies based on the packer’s given strategy ($a_{m2}$, $\beta$, $\theta$), the packer will determine its own optimal strategy $\left( {a_{m2}}^{*}, \beta^{*},\theta^{*} \right)$ based on the producer’s and retailer’s rational optimal feedback strategies $a_{s2}^{*}$ and $a_{r2}^{*}$ to satisfy profit maximization. Similarly, the optimal value function of the total present profit of the packer after time $t$ is

| $H_{m2}^{*}\left( q \right)= \max_{a_{m2}}\int_{t}^{\infty} e^{-\rho\tau}\{p_{m}D\left( q\left( \tau\right), \tau\right)-\frac{\mu_{m}}{2}\left[ a_{m2}\left( \tau\right) \right]^{2}-\beta\frac{\mu_{s}}{2}\left[ a_{s2}\left( \tau\right) \right]^{2}-\theta\frac{\mu_{r}}{2}\left[ a_{r2}\left( \tau\right) \right]^{2}\}d\tau$ | (A.48) |
| --- | --- |

Let the optimal value function of the total profit of the packer after time $t$ be

| $Y_{m2}\left( q \right)= \max_{a_{m2}}\int_{t}^{\infty} e^{-\rho(\tau-t)}\left\{ p_{r}D\left( q\left( \tau\right), \tau\right)-\frac{\mu_{m}}{2}\left[ a_{m2}\left( \tau\right) \right]^{2}-\beta\frac{\mu_{s}}{2}\left[ a_{s2}\left( \tau\right) \right]^{2}-\theta\frac{\mu_{r}}{2}\left[ a_{r2}\left( \tau\right) \right]^{2} \right\}d\tau$ | (A.49) |
| --- | --- |

Then the optimal value function of the total present profit of the packer after time $t$ can be expressed as

| $H_{m2}^{*}\left( q \right)= e^{-\rho t}Y_{m2}\left( q \right)$ | (A.50) |
| --- | --- |

Then $Y_{m2}\left( q \right)$ must satisfy the following HJB equation for all $q \geq0$.

| $\rho Y_{m2}\left( q \right)=\max_{a_{m2}} [p_{m}\left( D_{0}+\varphi q \right)-\frac{\mu_{m}}{2}{a_{m2}}^{2}-\beta\frac{\mu_{s}}{2}{a_{s2}}^{2}-\theta\frac{\mu_{r}}{2}{a_{r2}}^{2}+{Y_{m2}\left( q \right)}^{'} (\varepsilon a_{s2}+\delta a_{m2}+\gamma a_{r2}-\sigma q)]$ | (A.51) |
| --- | --- |

Substituting Eq. (A.46) and Eq. (A.47) into Eq. (A.51), we can obtain Eq. (A.52) by rectification.

| $\rho Y_{m2}\left( q \right)= {max}_{a_{m}}\left\{ p_{m}\left( D_{0}+\varphi q \right)-\frac{\mu_{m}}{2}{a_{m2}}^{2}-\frac{\beta\varepsilon^{2}{{Y_{s2}\left( q \right)}^{'}}^{2}}{2\mu_{s}\left( 1-\beta\right)^{2}}-\frac{\theta\gamma^{2}{{Y_{r2}\left( q \right)}^{'}}^{2}}{2\mu_{r}\left( 1-\theta\right)^{2}}+ {Y_{m2}\left( q \right)}^{'}\left[ \frac{\varepsilon^{2}{Y_{s2}\left( q \right)}^{'}}{\mu_{s}(1-\beta)}+\frac{\gamma^{2}{Y_{r2}\left( q \right)}^{'}}{\mu_{r}(1-\theta)}+\delta a_{m2}-\sigma q \right] \right\}$ | (A.52) |
| --- | --- |

Similarly, from the first-order condition, we can obtain $a_{m2}$, $\beta$ and $\theta$.

| $a_{m2}=\frac{\alpha{Y_{m2}\left( q \right)}^{'}}{\mu_{m}}$ | (A.53) |
| --- | --- |
| $\beta= \frac{2{Y_{m2}\left( q \right)}^{'}-{Y_{s2}\left( q \right)}^{'}}{2{Y_{m2}\left( q \right)}^{'}+{Y_{s2}\left( q \right)}^{'}}$ | (A.54) |
| $\theta= \frac{2{Y_{m2}\left( q \right)}^{'}-{Y_{r2}\left( q \right)}^{'}}{2{Y_{m2}\left( q \right)}^{'}+{Y_{r2}\left( q \right)}^{'}}$ | (A.55) |

Substituting Eq. (A.46), (A.47), (A.53), (A.54) and (A.55) into Eq. (A.44), (A.45) and (A.51).

| $\rho Y_{s2}\left( q \right)=(p_{s}\alpha-\sigma{Y_{s2}\left( q \right)}^{'}) q+p_{s}Q_{0}+\frac{\delta^{2}{Y_{m2}\left( q \right)}^{'}{Y_{s2}\left( q \right)}^{'}}{\mu_{m}}+\frac{\varepsilon^{2}{Y_{s2}\left( q \right)}^{'}(2{Y_{m2}\left( q \right)}^{'}+{Y_{s2}\left( q \right)}^{'})}{{4\mu}_{s}}+\frac{\gamma^{2}{Y_{s2}\left( q \right)}^{'}(2{Y_{m2}\left( q \right)}^{'}+{Y_{r2}\left( q \right)}^{'})}{2\mu_{r}}$ | (A.56) |
| --- | --- |
| $\rho Y_{r2}\left( q \right)=\left( p_{r}\varphi-\sigma{Y_{r2}\left( q \right)}^{'} \right)q+ p_{r}D_{0}+\frac{\delta^{2}{Y_{m2}\left( q \right)}^{'}{Y_{r2}\left( q \right)}^{'}}{\mu_{m}}+\frac{\varepsilon^{2}{Y_{r2}\left( q \right)}^{'}(2{Y_{m2}\left( q \right)}^{'}+{Y_{s2}\left( q \right)}^{'})}{{2\mu}_{s}}+\frac{\gamma^{2}{Y_{r2}\left( q \right)}^{'}(2{Y_{m2}\left( q \right)}^{'}+{Y_{r2}\left( q \right)}^{'})}{4\mu_{r}}$ | (A.57) |
| $\rho Y_{m2}\left( q \right)=(p_{m}\varphi-\sigma{Y_{m2}\left( q \right)}^{'}) q+p_{m}D_{0}+\frac{\delta^{2}{{Y_{m2}\left( q \right)}^{'}}^{2}}{2\mu_{m}}+\frac{\varepsilon^{2}{(2{Y_{m2}\left( q \right)}^{'}+{Y_{s2}\left( q \right)}^{'})}^{2}}{8\mu_{s}}+\frac{\gamma^{2}{(2{Y_{m2}\left( q \right)}^{'}+{Y_{r2}\left( q \right)}^{'})}^{2}}{8\mu_{r}}$ | (A.58) |

Noting that the differential Eq. (A.56), (A.57) and (A.58) are all first-order differential equations, it is presumed that the linear function on $q$ is the solution of the HJB equation.

| $Y_{s2}\left( q \right)= k_{4}q+b_{4}; Y_{r2}\left( q \right)= k_{5}q+b_{5}; Y_{m2}\left( q \right)= k_{6}q+b_{6}$ | (A.59) |
| --- | --- |

where $k_{4}$, $b_{4}$, $k_{5}$, $b_{5}$, $k_{6}$, $b_{6}$ are constants. The first order derivatives with respect to $q$ are found separately from Eq. (A.59).

| ${Y_{s2}\left( q \right)}^{'}= k_{4}; {Y_{r2}\left( q \right)}^{'}= k_{5}; {Y_{m2}\left( q \right)}^{'}= k_{6}$ | (A.60) |
| --- | --- |

Substituting Eq. (A.59) and (A.60) into Eq. (A.56), (A.57) and (A.58), respectively.

| $\rho\left( k_{4}q+b_{4} \right)= (p_{s}\alpha-\sigma k_{4}) q+p_{s}Q_{0}+\frac{\delta^{2}k_{4}k_{6}}{\mu_{m}}+\frac{\varepsilon^{2}k_{4}(2k_{6}+k_{4})}{{4\mu}_{s}}+\frac{\gamma^{2}k_{4}\left( 2k_{6}+k_{5} \right)}{2\mu_{r}}$ | (A.61) |
| --- | --- |
| $\rho{(k}_{5}q+b_{5})=\left( p_{r}\varphi-\sigma k_{5} \right)q+ p_{r}D_{0}+\frac{\delta^{2}k_{5}k_{6}}{\mu_{m}}+\frac{\varepsilon^{2}k_{5}(2k_{6}+k_{4})}{{2\mu}_{s}}+\frac{\gamma^{2}k_{5}\left( 2k_{6}+k_{5} \right)}{4\mu_{r}}$ | (A.62) |
| $\rho(k_{6}q+b_{6})=(p_{m}\varphi-\sigma k_{6}) q+p_{m}D_{0}+\frac{\delta^{2}{k_{6}}^{2}}{2\mu_{m}}+\frac{\varepsilon^{2}{(2k_{6}+k_{4})}^{2}}{8\mu_{s}}+\frac{\gamma^{2}{(2k_{6}+k_{5})}^{2}}{8\mu_{r}}$ | (A.63) |

Comparing the coefficients of similar terms on the left and right sides of Eq. (A.61), (A.62) and (A.63), we can obtain the equations system for $k_{4}$, $b_{4}$, $k_{5}$, $b_{5}$, $k_{6}$, $b_{6}$, as shown in Eq. (A.64).

| $\rho k_{4}= p_{s}\alpha-\sigma k_{4}$ | (A.64) |
| --- | --- |
| $\rho b_{4}= p_{s}Q_{0}+\frac{\delta^{2}k_{4}k_{6}}{\mu_{m}}+\frac{\varepsilon^{2}k_{4}(2k_{6}+k_{4})}{{4\mu}_{s}}+\frac{\gamma^{2}k_{4}\left( 2k_{6}+k_{5} \right)}{2\mu_{r}}$ |  |
| $\rho k_{5}= p_{r}\varphi-\sigma k_{5}$ |  |
| $\rho b_{5}= p_{r}D_{0}+\frac{\delta^{2}k_{5}k_{6}}{\mu_{m}}+\frac{\varepsilon^{2}k_{5}(2k_{6}+k_{4})}{{2\mu}_{s}}+\frac{\gamma^{2}k_{5}\left( 2k_{6}+k_{5} \right)}{4\mu_{r}}$ |  |
| $\rho k_{6}= p_{m}\varphi-\sigma k_{6}$ |  |
| $\rho b_{6}= p_{m}D_{0}+\frac{\delta^{2}{k_{6}}^{2}}{2\mu_{m}}+\frac{\varepsilon^{2}{(2k_{6}+k_{4})}^{2}}{8\mu_{s}}+\frac{\gamma^{2}{(2k_{6}+k_{5})}^{2}}{8\mu_{r}}$ |  |

Solving the equations system (A.64), we can get the results of $k_{4}$*,* $b_{4}$*,* $k_{5}$*,* $b_{5}$*,* $k_{6}$*,* $b_{6}$.

| $k_{4}= \frac{p_{s}\alpha}{\rho+\sigma}$ | (A.65) |
| --- | --- |
| $b_{4}= \frac{p_{s}Q_{0}}{\rho}+\frac{\delta^{2}\alpha\varphi p_{s}p_{m}}{\rho\mu_{m}{(\rho+\sigma)}^{2}}+\frac{\varepsilon^{2}\partial p_{s}(2\varphi p_{m}+\alpha p_{s})}{4\rho\mu_{s}{(\rho+\sigma)}^{2}}+\frac{\gamma^{2}\alpha\varphi p_{s}(2p_{m}+p_{r})}{2\rho\mu_{r}{(\rho+\sigma)}^{2}}$ |  |
| $k_{5}= \frac{p_{r}\varphi}{\rho+\sigma}$ |  |
| $b_{5}=\frac{p_{r}D_{0}}{\rho}+\frac{\delta^{2}\varphi^{2}p_{r}p_{m}}{\rho\mu_{m}{(\rho+\sigma)}^{2}}+\frac{\varepsilon^{2}\varphi p_{r}(2\varphi p_{m}+\alpha p_{s})}{2\rho\mu_{s}{(\rho+\sigma)}^{2}}+\frac{\gamma^{2}\alpha\varphi p_{s}(2p_{m}+p_{r})}{4\rho\mu_{r}{(\rho+\sigma)}^{2}}$ |  |
| $k_{6}= \frac{p_{m}\varphi}{\rho+\sigma}$ |  |
| $b_{6}=\frac{p_{m}D_{0}}{\rho}+\frac{\delta^{2}\varphi^{2}{p_{m}}^{2}}{2\rho\mu_{m}{(\rho+\sigma)}^{2}}+\frac{\varepsilon^{2}{(2\varphi p_{m}+\partial p_{s})}^{2}}{8\rho\mu_{s}{(\rho+\sigma)}^{2}}+\frac{\gamma^{2}\varphi^{2}{(2p_{m}+p_{r})}^{2}}{8\rho\mu_{r}{(\rho+\sigma)}^{2}}$ |  |

Substituting Eq. (A.65) into Eq. (A.60).

| ${Y_{s2}\left( q \right)}^{'}= \frac{p_{s}\alpha}{\rho+\sigma}; {Y_{r2}\left( q \right)}^{'}= \frac{p_{r}\varphi}{\rho+\sigma}; {Y_{m2}\left( q \right)}^{'}= \frac{p_{m}\varphi}{\rho+\sigma}$ | (A.66) |
| --- | --- |

Substituting Eq. (A.66) into Eq. (A.46), (A.47), (A.53), (A.54) and (A.55).

| ${a_{s2}}^{*}=\frac{\varepsilon(2\varphi p_{m}+\alpha p_{s})}{2\mu_{s}\left( \rho+\sigma\right)}$ | (A.67) |
| --- | --- |
| ${a_{r2}}^{*}=\frac{\gamma\varphi(2p_{m}+p_{r})}{2\mu_{r}(\rho+\sigma)}$ | (A.68) |
| ${a_{m2}}^{*}=\frac{\delta\varphi p_{m}}{\mu_{m}(\rho+\sigma)}$ | (A.69) |
| $\beta= \left\{ \begin{aligned} \frac{2\varphi p_{m}-\alpha p_{s}}{2\varphi p_{m}+\alpha p_{s}} \\ 0 \end{aligned}{when \varphi p_{m}\geq\frac{\alpha p_{s}}{2} \atop when \varphi p_{m}<\frac{\alpha p_{s}}{2}} \right.$ | (A.70) |
| $\theta= \left\{ \begin{aligned} \frac{2p_{m}-p_{r}}{2p_{m}+p_{r}} \\ 0 \end{aligned}{when p_{m}\geq\frac{p_{r}}{2} \atop when p_{m}<\frac{p_{r}}{2}} \right.$ | (A.71) |

Substituting the equations system (A.65) into Eq. (A.59), we can derive the optimal value functions of the total profit equivalent value of the producer, retailer and packer.

| ${Y_{s2}\left( q \right)}^{*}= \frac{p_{s}\alpha}{\rho+\sigma}q+ \frac{p_{s}Q_{0}}{\rho}+\frac{\delta^{2}\alpha\varphi p_{s}p_{m}}{\rho\mu_{m}{(\rho+\sigma)}^{2}}+\frac{\varepsilon^{2}\alpha p_{s}(2\varphi p_{m}+\alpha p_{s})}{4\rho\mu_{s}{(\rho+\sigma)}^{2}}+\frac{\gamma^{2}\alpha\varphi p_{s}(2p_{m}+p_{r})}{2\rho\mu_{r}{(\rho+\sigma)}^{2}}$ | (A.72) |
| --- | --- |
| ${Y_{r2}\left( q \right)}^{*}= \frac{p_{r}\varphi}{\rho+\sigma}q+\frac{p_{r}D_{0}}{\rho}+\frac{\delta^{2}\varphi^{2}p_{r}p_{m}}{\rho\mu_{m}{(\rho+\sigma)}^{2}}+\frac{\varepsilon^{2}\varphi p_{r}(2\varphi p_{m}+\alpha p_{s})}{2\rho\mu_{s}{(\rho+\sigma)}^{2}}+\frac{\gamma^{2}\varphi^{2}p_{r}(2p_{m}+p_{r})}{4\rho\mu_{r}{(\rho+\sigma)}^{2}}$ | (A.73) |
| ${Y_{m2}\left( q \right)}^{*}= \frac{p_{m}\varphi}{\rho+\sigma}q+\frac{p_{m}D_{0}}{\rho}+\frac{\delta^{2}\varphi^{2}{p_{m}}^{2}}{2\rho\mu_{m}{(\rho+\sigma)}^{2}}+\frac{\varepsilon^{2}{(2\varphi p_{m}+\alpha p_{s})}^{2}}{8\rho\mu_{s}{(\rho+\sigma)}^{2}}+\frac{\gamma^{2}\varphi^{2}\left( 2p_{m}+p_{r} \right)^{2}}{8\rho\mu_{r}\left( \rho+\sigma\right)^{2}}$ | (A.74) |

Substituting Eq. (A.72), (A.73), (A.74) into Eq. (A.42), (A.43), (A.50), we can obtain the optimal value functions of the total present profit of the producer, retailer and packer.

| $H_{s2}^{*}\left( q \right)= e^{-\rho t}{Y_{s2}\left( q \right)}^{*}$ | (A.75) |
| --- | --- |
| $H_{r2}^{*}\left( q \right)= e^{-\rho t}{Y_{r2}\left( q \right)}^{*}$ | (A.76) |
| $H_{m2}^{*}\left( q \right)= e^{-\rho t}{Y_{m2}\left( q \right)}^{*}$ | (A.77) |

Then, the trajectory of the product emissions reduction can be solved by substituting Eq. (A.67), (A.68) and (A.69) into the state equation Eq. (1).

| $q\left( t \right)= \omega-(\omega-q_{0})e^{-\sigma t}$ | (A.78) |
| --- | --- |

where $\omega= \frac{\delta^{2}\varphi p_{m}}{\sigma\mu_{m}(\rho+\sigma)}+\frac{\varepsilon^{2}(2\varphi p_{m}+\alpha p_{s})}{2{\sigma\mu}_{s}\left( \rho+\sigma\right)}+\frac{\gamma^{2}\varphi(2p_{m}+p_{r})}{2\sigma\mu_{r}(\rho+\sigma)}$. Thus, Proposition 2 is proven.

Finally, let ${H_{2}}^{*}(q)$ denote the present profit of the whole agrifood value chain.

| ${H_{2}}^{*}(q)=H_{s2}^{*}\left( q \right)+H_{m2}^{*}\left( q \right)+H_{r2}^{*}\left( q \right)$ | (A.79) |
| --- | --- |

**Proof of Proposition 3**

As in Section 3.2, the optimal value functions of the total present profit of the agrifood value chain after time $t$ is

| ${H_{w}^{c}}^{*}\left( q(t) \right)=\max_{a_{m}^{c},{a_{s}^{c}, a}_{r}^{c}}\int_{t}^{\infty} e^{-\rho\tau}\left\{ \left( p_{m}+p_{r} \right)D\left( q\left( \tau\right), \tau\right)+p_{s}Q\left( q\left( \tau\right),\tau\right)-\frac{\mu_{m}}{2}\left[ a_{m}^{c}\left( \tau\right) \right]^{2}-\frac{\mu_{s}}{2}\left[ a_{s}^{c}\left( \tau\right) \right]^{2}-\frac{\mu_{r}}{2}\left[ a_{r}^{c}\left( \tau\right) \right]^{2} \right\}d\tau$ | (A.80) |
| --- | --- |

Let the optimal value function of the total profit of the value chain after time $t$ be

| $Y_{w}\left( q \right)= \max_{a_{m}^{c},{a_{s}^{c}, a}_{r}^{c}}\int_{t}^{\infty} e^{-\rho(\tau-t)}\left\{ \left( p_{m}+p_{r} \right)D\left( q\left( \tau\right), \tau\right)+p_{s}Q\left( q\left( \tau\right),\tau\right)-\frac{\mu_{m}}{2}\left[ a_{m}^{c}\left( \tau\right) \right]^{2}-\frac{\mu_{s}}{2}\left[ a_{s}^{c}\left( \tau\right) \right]^{2}-\frac{\mu_{r}}{2}\left[ a_{r}^{c}\left( \tau\right) \right]^{2} \right\}d\tau$ | (A.81) |
| --- | --- |

Then the optimal value function of the total present profit after time $t$ can be expressed as

| ${H_{w}^{c}}^{*}\left( q \right)= e^{-\rho t}Y_{w}\left( q \right)$ | (A.82) |
| --- | --- |

Then $Y_{w}\left( q \right)$ must satisfy the following HJB equation for all $q \geq0$.

| $\rho Y_{w}\left( q \right)=\max_{a_{m}^{c},{a_{s}^{c}, a}_{r}^{c}} [\left( p_{m}+p_{r} \right)\left( D_{0}+\varphi q \right)+p_{s}\left( Q_{0}+\alpha q \right)-\frac{\mu_{m}}{2}{a_{m}^{c}}^{2}-\frac{\mu_{s}}{2}{a_{s}^{c}}^{2}-\frac{\mu_{r}}{2}{a_{r}^{c}}^{2}+{Y_{w}\left( q \right)}^{'} (\varepsilon a_{s}^{c}+\delta a_{m}^{c}+\gamma a_{r}^{c}-\sigma q)]$ | (A.83) |
| --- | --- |

The optimal emissions reduction strategies of the packer, producer and retailer under the cooperative mechanism can be solved from the first-order conditions.

| $a_{m}^{c}= \frac{\delta{Y_{w}\left( q \right)}^{'}}{\mu_{m}}$ | (A.84) |
| --- | --- |
| $a_{s}^{c}=\frac{\varepsilon{Y_{w}\left( q \right)}^{'}}{\mu_{s}}$ | (A.85) |
| $a_{r}^{c}=\frac{\gamma{Y_{w}\left( q \right)}^{'}}{\mu_{r}}$ | (A.86) |

Substituting Eq. (A.84), (A.85) and (A.86) into Eq. (A.83).

| $\rho Y_{w}\left( q \right)=\left( \left( p_{m}+p_{r} \right)\varphi-{{\sigma Y}_{w}\left( q \right)}^{'}+\alpha p_{s} \right)q+\left( p_{m}+p_{r} \right)D_{0}+p_{s}Q_{0}+\left( \frac{\delta^{2}}{2\mu_{m}}+\frac{\varepsilon^{2}}{2\mu_{s}}+\frac{\gamma^{2}}{2\mu_{r}} \right){{Y_{w}\left( q \right)}^{'}}^{2}$ | (A.87) |
| --- | --- |

From Eq. (A.87), the linear function on $q$ is the solution of this HJB equation.

| $Y_{w}\left( q \right)=kq+b$ | (A.88) |
| --- | --- |

where k and b are constants. Substituting Eq. (A.88) and its first-order derivative with respect to $q$ into Eq. (A.87), and sorting and comparing coefficients of similar terms on the left and right, we can solve for k and b.

| $k= \frac{\left( p_{m}+p_{r} \right)\varphi+p_{s}\alpha}{\rho+\sigma}$ | (A.89) |
| --- | --- |
| $b= \frac{\left( p_{m}+p_{r} \right)D_{0}+p_{s}Q_{0}}{\rho}+\frac{\left[ \left( p_{m}+p_{r} \right)\varphi+p_{s}\alpha\right]^{2}}{2\rho\left( \rho+\sigma\right)^{2}}\left( \frac{\delta^{2}}{\mu_{m}}+\frac{\varepsilon^{2}}{\mu_{s}}+\frac{\gamma^{2}}{\mu_{r}} \right)$ |  |

Substituting Eq. (A.89) into Eq. (A.84), (A.85) and (A.86). We can obtain the feedback equilibrium strategies of the packer, producer, and retailer on the emissions reduction efforts in the cooperative game of emissions reduction.

| ${a_{m}^{c}}^{*}= \frac{\delta\left[ \left( p_{m}+p_{r} \right)\varphi+p_{s}\alpha\right]}{\mu_{m}(\rho+\sigma)}$ | (A.90) |
| --- | --- |
| ${a_{s}^{c}}^{*}=\frac{\varepsilon\left[ \left( p_{m}+p_{r} \right)\varphi+p_{s}\alpha\right]}{\mu_{s}(\rho+\sigma)}$ | (A.91) |
| ${a_{r}^{c}}^{*}=\frac{\gamma\left[ \left( p_{m}+p_{r} \right)\varphi+p_{s}\alpha\right]}{\mu_{r}(\rho+\sigma)}$ | (A.92) |

Substituting Eq. (A.89) into Eq. (A.88).

| ${Y_{w}\left( q \right)}^{*}=\frac{\left( p_{m}+p_{r} \right)\varphi+p_{s}\alpha}{\rho+\sigma}q+ \frac{\left( p_{m}+p_{r} \right)D_{0}+p_{s}Q_{0}}{\rho}+\frac{\left[ \left( p_{m}+p_{r} \right)\varphi+p_{s}\alpha\right]^{2}}{2\rho\left( \rho+\sigma\right)^{2}}\left( \frac{\delta^{2}}{\mu_{m}}+\frac{\varepsilon^{2}}{\mu_{s}}+\frac{\gamma^{2}}{\mu_{r}} \right)$ | (A.93) |
| --- | --- |

Substituting Eq. (A.93) into Eq. (A.82), we can obtain the optimal value function of the total present profit of the agrifood value chain system.

| ${H_{w}^{c}}^{*}\left( q \right)= e^{-\rho t}{Y_{w}\left( q \right)}^{*}$ | (A.94) |
| --- | --- |

Then, the trajectory of the product emissions reduction can be solved by substituting Eq. (A.90), (A.91) and (A.92) into the state equation Eq. (1).

| ${{q\left( t \right)}^{c}}^{*}= v-(v-q_{0})e^{-\sigma t}$ | (A.95) |
| --- | --- |

where $v = \frac{\delta^{2}\left[ \left( p_{m}+p_{r} \right)\varphi+p_{s}\alpha\right]}{\sigma\mu_{m}(\rho+\sigma)}+\frac{\varepsilon^{2}\left[ \left( p_{m}+p_{r} \right)\varphi+p_{s}\alpha\right]}{{\sigma\mu}_{s}\left( \rho+\sigma\right)}+\frac{\gamma^{2}\left[ \left( p_{m}+p_{r} \right)\varphi+p_{s}\alpha\right]}{\sigma\mu_{r}(\rho+\sigma)}$. Thus, Proposition 3 is proven.
